# An efficient discrete Chebyshev polynomials strategy for tempered time fractional nonlinear Schrödinger problems^☆^

**DOI:** 10.1016/j.jare.2024.11.014

**Published:** 2024-11-16

**Authors:** Mohammad Hossein Heydari, Dumitru Baleanu

**Affiliations:** a Department of Mathematics, Shiraz University of Technology, Shiraz, Iran; b Department of Computer Science and Mathematics, Lebanese American University, Beirut 13-5053, Lebanon

**Keywords:** Tempered time fractional nonlinear Schrödinger, Tempered time fractional coupled nonlinear Schrödinger equations, Orthonormal discrete Chebyshev polynomials, Operational matrices

## Abstract

****Introduction**:**

An interesting type of fractional derivatives that has received widespread attention in recent years is the tempered fractional derivatives. These fractional derivatives are a generalization of the well-known fractional derivatives, such as Caputo and Riemann–Liouville. In fact, these derivatives are obtained by multiplying the expressed fractional derivatives by an exponential factor. These fractional derivatives have an additional parameter called λ such that in the case of λ=0, the classical Caputo or Riemann–Liouville fractional derivative is obtained.

****Objectives**:**

Employing the Caputo tempered fractional derivative to define time fractional nonlinear Schrödinger equation and a coupled system of nonlinear Schrödinger equations. Applying the orthonormal discrete Chebyshev polynomials (ODCPs) to solve these problems. For this purposes, the operational matrices of ordinary and tempered fractional derivatives of the ODCPs are obtained.

****Methods**:**

By representing the problem’s solutions in terms of the ODCPs (with some unknown coefficients) and exploiting the expressed operational matrices, along with the collocation strategy, two systems of nonlinear algebraic equations are derived. By solving these systems, the expressed coefficients, and subsequently the solution of the main fractional problems are obtained.

****Results**:**

Some numerical examples are investigated to acknowledge the high accuracy of the designed approaches.

****Conclusion**:**

The tempered fractional derivative in the Caputo form is utilized to define the time fractional nonlinear Schrödinger equation and a coupled system of nonlinear Schrödinger equations. The ODCPs are used to design a numerical strategy for these problems. To this purpose, some operational matrices for these polynomials are obtained. In the designed procedures, the problem’s solution are obtained by solving an algebraic system of equations. These systems are obtained by approximating the solution with the ODCPs and employing the expressed matrix relationships, along with the collocation technique. Some examples are presented to check the validity of the developed algorithms. The reported results acknowledged the high accuracy of the designed schemes.

## Highlights

• Tempered time fractional nonlinear Schrodinger and coupled Schrodinger equations are defined.
• The orthonormal discrete Chebyshev polynomials (DCPs) are used to solve the introduced problems.
• Operational matrix of tempered fractional integral of the orthonormal DCPs is obtained.
• Operational matrix of the second order derivative of the orthonormal DCPs is extracted.
• The accuracy of the proposed method is investigated in some numerical examples.

## Graphical abstract

**Figure:**
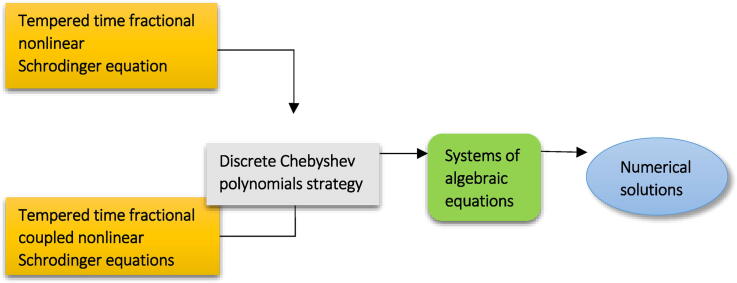

## Introduction

In recent decades, the topic of fractional calculus (derivative and integral of arbitrary order) has been one of the hot research topics for researchers in engineering and science fields. We remind that the more degree of freedom of fractional derivatives and integrals compared to ordinary ones, together with their memory property and non-locality, have increased the capabilities of these operators [1]. This subject has been investigated from different aspects. Some of the studies done in this field are related to the application of fractional derivatives in modeling important practical problems. In this case, their recent applications in viscoelastic models [2], falling objects [3], prediction of tumor growth [4], duffing oscillator [5], dynamics of HIV/AIDS transmission [6], dynamics of reversible enzymatic reaction [7], and modeling of Salmonella bacterial infection [8] can be mentioned. A large number of recent researches in the field of fractional calculus have been related to providing effective numerical methods to find the solution of fractional differential equations (equations containing at least one term with a fractional derivative). The most famous numerical technique used for such problems are finite volume method [9], finite difference method [10], finite element method [11], spectral method [12], meshless method [13], wavelet method [14], time stepping schemes jointed with the spectral Chebyshev-Legendre collocation method [15], etc. Another number of studies done in relation to fractional calculus are related to providing new definitions of fractional integrals and derivatives. Some important definitions of fractional derivatives and integrals can be seen in reference [16]. An interesting class of fractional derivatives that has received widespread attention in recent years is the tempered fractional derivatives [17]. These kinds of fractional derivative are a generalization of the well-known fractional derivatives, such as Caputo and Riemann–Liouville. In fact, these derivatives are obtained by multiplying the expressed fractional derivatives by an exponential factor [18]. These fractional derivatives have an additional parameter called λ such that in the case of λ=0, the classical Caputo or Riemann–Liouville fractional derivative is obtained [18]. It is worth noting that the tempered fractional derivatives have many advantages over the traditional fractional derivatives, such as

• Providing a more flexible approach to modeling phenomena that exhibit both local and non-local dynamics, which classical fractional derivatives may not capture as effectively.
• Having a great ability in describing transitions between normal and anomalous diffusions within finite time or bounded space domains, which is significant for accurate modeling in mathematical physics.
• Facilitating in the development of finite difference schemes for solving tempered fractional diffusion equations (in the case of tempered fractional derivatives by tempered fractional difference quotients).
• Having a suitable structure to extend traditional concepts by incorporating an exponential factor, which can lead to new insights and applications in various fields of science and engineering.

These advantages make tempered fractional derivatives a powerful tool for researchers and practitioners in fields that require sophisticated mathematical modeling and analysis [19], [20]. In recent years, many studies have been conducted on these fractional derivatives, due to their wide applications in physics [21], geophysics [22], finance [23], wind spreed date [17], poroelasticity [24], etc. The most important challenge related to the problems involved with this type of derivatives is to find their solution. In recent years, numerical methods have been used as an effective tool to solve such problems. Some of the numerical methods used in this regard are finite difference and finite element methods [25], Riesz basis Galerkin approach [26], piecewise polynomials collocation method [27], backward Euler technique combined with Galerkin finite element method [28], Legendre polynomials method [29], matrix splitting preconditioning technique[30] and Crank-Nicolson ADI finite difference scheme [31].

The nonlinear Schrödinger equation is a well-known and well-studied differential equation in physics [32] that explains the quantum behavior transformation of incoordinate physical systems with respect to time. In fact, this equation describes the conversion of a low rate change wave package in a weak system with highly scattered mediums [33]. Fractional forms of the nonlinear Schrödinger equation possess useful applications in quantum mechanics [34]. In recent years, numerical approaches for the fractional nonlinear Schrödinger equation (generated using different types of fractional derivatives) have received more attention. Some of these methods are piecewise Jacobi functions method [35], reproducing kernel method [36], linearized compact ADI techniques [37], neural network method [38], fourth-order accurate conservative method [39], finite difference method [40], hybrid clique functions method [41], splitting conservative difference method [42], conservative exponential relaxation technique [43], weighted average nonstandard finite difference method [32], [44], etc.

The fractional coupled nonlinear Schrödinger equations arise in many problems related to optics and hydrodynamics [45]. These coupled equations describe the interaction between two waves with different frequencies [45]. One of the main challenges in facing such fractional systems is finding their solutions. In recent years, various numerical algorithms have been used to solve the fractional coupled nonlinear Schrödinger equations. Some of these methods are linearized Crank-Nicolson method [46], linearized conservative finite element technique [47], compact finite difference method [48], Legendre-Galerkin spectral method [49], etc.

The lack of adequate research on the tempered fractional form of the nonlinear Schrödinger problems made us interested in introducing such a form of these problems in this work. On the other hand, since it is not possible to solve the presented problems with analytical methods, it is important to solve them with a suitable numerical method. Therefore, in the continuation of this research, we focus on the numerical solution of the following tempered fractional problems:

**(I)** Tempered time fractional nonlinear Schrödinger equation

(1) iDtα,λ0cΨ+θ∂xxΨ+σΨ2Ψ+ηΨ=f.

**(II)** Tempered time fractional coupled nonlinear Schrödinger equations

(2) iDtα,λ0cΨ+θ∂xxΨ+σ1Ψ2+σ2Φ2Ψ+η1Ψ+η2Φ=g,iDtα,λ0cΦ+θ∂xxΦ+σ1Ψ2+σ2Φ2Φ+η1Φ+η2Ψ=h.

In the above problem, (x,t) belongs to the domain $[0,1]\times [0,1],\partial_{\mathrm{xx}}=\partial^{2}/\partial x^{2}$ denotes the second order partial derivative, $i=\sqrt{-1}$ indicates the imaginary unit, Ψ=Ψ(x,t) and Φ=Φ(x,t) are unknown complex solutions, f=f(x,t),g=g(x,t) and h=h(x,t) are given complex functions, $\theta ,\sigma ,\sigma_{1}$ and $\sigma_{2}$ are non-negative real numbers, $\eta =\eta (x),\eta_{1}=\eta_{1}(x)$ and $\eta_{2}=\eta_{2}(x)$ are given real functions. Moreover, 0<α⩽1 and λ⩾0 are given real numbers, and Dtα,λ0cΨ denotes the tempered time fractional derivative of order α in the Caputo type (more details about this kind of fractional derivative are given in the next section). An effective strategy for solving fractional differential equations is to convert the problem under investigation into a simpler algebraic problem by using appropriate basis functions so that the solution to the original problem can be obtained by solving this algebraic problem. An effective and smart choice for basis functions can be polynomials. Because it is easy to calculate their fractional derivative with a low computational cost. In addition, if the solution of the problem is sufficiently differentiable, the results of the numerical method made by these polynomials have exponential (spectral) accuracy. Note that basis polynomials are divided into continuous and discrete. In the continuous expansion of a known function (by using continuous polynomials), the expansion coefficients are calculated by integration (in most cases in an approximate way), while in the discrete expansion (by using discrete polynomials), the corresponding coefficients are precisely calculated by a finite series. Due to the stated reasons, discrete polynomials have been given much attention in recent years to solve various fractional problems. For example, the discrete Legendre polynomials are used in [50] for fractional reaction–diffusion equations. The discrete Hahn polynomials are utilized in [51] for fractional Rayleigh-Stokes equation. In [52], the discrete Chebyshev polynomials are applied for fractional integro-differential equations. The discrete Chebyshev polynomials, as a well-known family of the discrete polynomials, have been introduced for the first time in [53]. The orthonormal form of these polynomials have been defined in [54]. In this paper, the orthonormal discrete Chebyshev polynomials (ODCPs) have been used as the appropriate basis functions to solve the problems introduced above. For this purpose, first, some operational matrices related to these polynomials (including ordinary and tempered fractional derivatives) are obtained. By approximating the solution of the mentioned problems via these polynomials (for the spatial and temporal variables) and exploiting the expressed matrices, along with the collocation method, the desired algebraic problems are obtained. To show the accuracy of the presented methods, some numerical examples have been examined. This work is arranged as follows: Some preliminaries about tempered fractional calculus are reviewed in Section 2. The desired basis functions are introduced in Section 3. The derivative operational matrices are derived in Section 4. The established schemes are explained in Section 5. Numerical investigations are done in Section 6. Conclusion of this study is explained in Section 7.

## Preliminaries

In this section, we briefly review some preliminaries about tempered fractional calculus.

Definition 1 ([1]) Let *u* is a suitably smooth function on [0,T] and m^-1<β⩽m^∈Z+ is a given number. The Caputo fractional derivative of order β of the function *u* is given by

(2.1) Dtβ0cu(t)=1Γ(m^-β)∫0t(t-s)m^-β-1u(m^)(s)ds,m^-1<β<m^,u(m^)(t),β=m^,

for 0<t⩽T.

Property 1 ([1]) For m^-1<β<m^ and ξ>m^-1, we have

(2.2) Dtβ0ctξ=Γ(ξ+1)Γ(ξ-β+1)tξ-β.

As a direct consequence, for k∈N∪{0}, we obtain

(2.3) Dtβ0ctk=0,k<m^,k!Γ(k-β+1)tk-β,k⩾m^.

Definition 2 ([17]) Let *u* is a suitably smooth function on [0,T],λ⩾0 is a given constant and m^-1<β⩽m^∈Z+ is a given number. The tempered fractional derivative in the Caputo type of order β and fixed parameter λ of the function *u* is given by

(2.4) $$D_{t}^{\beta ,\lambda }u\left(t\right)=e^{-\lambda t}D_{t}^{\beta }\left(e^{\lambda t}u\left(t\right)\right)\;\;\;\;\;\;\;\;\;\;\;\;\;\;\;=\left\{\begin{array}{cc} \frac{e^{-\lambda t}}{\Gamma \left(\hat{m}-\beta \right)}\int_{0}^{t}{\left(t-s\right)}^{\hat{m}-\beta -1}\frac{d^{\hat{m}}\left(e^{\lambda s}u\left(s\right)\right)}{{\mathrm{ds}}^{\hat{m}}}\mathrm{ds}, & \hat{m}-1<\beta <\hat{m},\\ \frac{e^{-\lambda t}d^{\hat{m}}\left(e^{\lambda t}u\left(t\right)\right)}{{\mathrm{dt}}^{\hat{m}}}, & \beta =\hat{m}\text{.} \end{array}\right)$$

Property 2 Based on the Property 1, for m^-1<β<m^ and ξ>m^-1, we have

(2.5) Dtβ,λ0ce-λttξ=Γ(ξ+1)Γ(ξ-β+1)e-λttξ-β.

As a direct consequence, for k∈N∪{0}, we obtain

(2.6) Dtβ,λ0ce-λttk=0,k<m^,k!Γ(k-β+1)e-λttk-β,k⩾m^.

## Orthonormal discrete Chebyshev polynomials

For a given positive integer *m*, a set with (m+1) elements of the ODCPs defined on [0,1], can be created by the formula [54]

(3.1) $$C_{m,i}(x)=\overset{i}{\underset{k=0}{\sum }}\overset{k}{\underset{r=0}{\sum }}a_{m,i,k,r}x^{r},i=0,1,\ldots ,m,$$

where

(3.2) am,i,k,r=(-1)kmrρm,ii+kim-ki-kk!Sk(r),ρm,i=(i+m+1)!(2i+1)(m-i)!(i!)2,

and

Skr=∑l=0k-r-1lk+l-1k+l-r2k-rk-l-rsk+l-rl,skr=1r!∑l=0r-1lrlr-lk.

Notice that the set ${\left\{C_{m,i}(x)\right\}}_{i=0}^{m}$ creates a complete set of orthonormal polynomials defined on [0,1] using the inner product

$$u,v=\overset{m}{\underset{l=0}{\sum }}u\left(\frac{l}{m}\right)v\left(\frac{l}{m}\right)\text{.}$$

Thus, any piecewise continuous function *u* defined on [0,1] can be presented via these orthonormal polynomials as

(3.3) $$u(x)≃\overset{m}{\underset{i=0}{\sum }}u_{i}C_{m,i}(x)≔U_{m}^{T}\Omega_{m}(x),$$

where $U_{m}={\left[u_{0}\;u_{1}\ldots u_{m}\right]}^{T}$ with

(3.4) $$u_{i}=\overset{m}{\underset{l=0}{\sum }}u\left(\frac{l}{m}\right)C_{m,i}\left(\frac{l}{m}\right),$$

and

(3.5) $$\Omega_{m}(x)={\left[C_{m,0}(x)\;C_{m,1}(x)\ldots C_{m,m}(x)\right]}^{T}\text{.}$$

Likewise, for any piecewise continuous function *v* defined on [0,1]×[0,1], the following approximation can be considered:

(3.6) $$v(x,t)≃\overset{m}{\underset{i=0}{\sum }}\overset{n}{\underset{j=0}{\sum }}v_{\mathrm{ij}}C_{m,i}(x)C_{n,j}(t)≔\Omega_{m}^{T}(x)V_{m\times n}\Omega_{n}(t),$$

where $V_{m\times n}=\left[v_{\mathrm{ij}}\right]$ with $v_{\mathrm{ij}}=\sum_{l=0}^{m}\sum_{\overset{¯}{l}=0}^{n}v\left(\frac{l}{m},\frac{\overset{¯}{l}}{n}\right)C_{m,i}\left(\frac{l}{m}\right)C_{n,j}\left(\frac{\overset{¯}{l}}{n}\right)$ for 0⩽i⩽m and 0⩽j⩽n.

## New operational matrices

In this section, we explain the procedure of calculating the second order derivative matrix and tempered fractional derivative matrix of the ODCPs. These matrices will be used in the proposed methods. Notice that these matrices reduce the computations in the proposed methods.

Theorem 1 For the vector $\Omega_{m}(x)$ given in (3.5), we have

(4.1) $$\frac{d^{2}\Omega_{m}(x)}{{\mathrm{dx}}^{2}}=D_{m}^{\left(2\right)}\Omega_{m}(x),$$

where $D_{m}^{\left(2\right)}=\left[d_{\mathrm{ij}}^{\left(2\right)}\right]$ for 0⩽i,j⩽m and

$$d_{\mathrm{ij}}^{\left(2\right)}=\left\{\begin{array}{cc} \sum_{l=0}^{m}\left(\sum_{k=2}^{i}\sum_{r=2}^{k}r\left(r-1\right)a_{m,i,k,r}{\left(\frac{l}{m}\right)}^{r-2}\right)C_{m,j}\left(\frac{l}{m}\right), & i=2,3,\ldots ,m,\;\;\;\;\;\;\;\;\;\;\;\;\;\;\;\;\;\;\;\;\;j=0,1,\ldots i-2,\\ 0, & \mathrm{otherwise}\text{.} \end{array}\right)$$

such that the coefficients $a_{m,i,k,r}$ have already been introduced in (3.2).

Proof Based on the formula given in (3.1), we get

$$\frac{d^{2}C_{m,i}(x)}{{\mathrm{dx}}^{2}}=\left\{\begin{array}{ccc} 0, & & i=0,1,\\ \overset{i}{\underset{k=2}{\sum }}\overset{k}{\underset{r=2}{\sum }}r(r-1)a_{m,i,k,r}x^{r-2}, & & i=2,3,\ldots ,m\text{.} \end{array}\right)$$

Thus, from (3.5) and the above relation, we have

$$\frac{d^{2}\Omega_{m}(x)}{{\mathrm{dx}}^{2}}={\left[0\;\;0\;\;\frac{d^{2}C_{m,2}(x)}{{\mathrm{dx}}^{2}}\;\;\frac{d^{2}C_{m,3}(x)}{{\mathrm{dx}}^{2}}\ldots \frac{d^{2}C_{m,m}(x)}{{\mathrm{dx}}^{2}}\right]}^{T}\text{.}$$

The non-zero components in the above vector can be represented by the ODCPs as

$$\frac{d^{2}C_{m,i}(x)}{{\mathrm{dx}}^{2}}=\overset{m}{\underset{j=0}{\sum }}d_{\mathrm{ij}}^{\left(2\right)}C_{m,j}(x),$$

where

$$d_{\mathrm{ij}}^{\left(2\right)}=\overset{m}{\underset{l=0}{\sum }}{\left(\left(\frac{d^{2}C_{m,i}(x)}{{\mathrm{dx}}^{2}}\right)\right|}_{x=\frac{l}{m}}C_{m,j}\left(\frac{l}{m}\right)\text{.}$$

The above result together with the formula expressed in (3.1), give

$$d_{\mathrm{ij}}^{\left(2\right)}=\overset{m}{\underset{l=0}{\sum }}\left(\overset{i}{\underset{k=2}{\sum }}\overset{k}{\underset{r=2}{\sum }}r(r-1)a_{m,i,k,r}{\left(\frac{l}{m}\right)}^{r-2}\right)C_{m,j}\left(\frac{l}{m}\right)\text{.}$$

It also should be noted that that the orthonormal property of the ODCPs yield that $d_{\mathrm{ij}}^{\left(2\right)}=0$ for j⩾i-1. Thus, we get

$$\frac{d^{2}\Omega_{m}(x)}{{\mathrm{dx}}^{2}}=\left(\begin{array}{cccccccc} 0 & 0 & 0 & \ldots & 0 & 0 & 0 & 0\\ 0 & 0 & 0 & \ldots & 0 & 0 & 0 & 0\\ d_{20}^{\left(2\right)} & 0 & 0 & \ldots & 0 & 0 & 0 & 0\\ d_{30}^{\left(2\right)} & d_{31}^{\left(2\right)} & 0 & \ldots & 0 & 0 & 0 & 0\\ \vdots & \vdots & \vdots & \ldots & \vdots & \vdots & \vdots & \vdots \\ d_{m0}^{\left(2\right)} & d_{m1}^{\left(2\right)} & d_{m2}^{\left(2\right)} & \ldots & d_{\mathrm{mm}-3}^{\left(2\right)} & d_{\mathrm{mm}-2}^{\left(2\right)} & 0 & 0 \end{array}\right)\Omega_{m}(x)≔D_{m}^{\left(2\right)}\Omega_{m}(x),$$

which ends the proof.

As it can be seen above, the entries of the first two rows and the first two columns of the matrix $D_{m}^{\left(2\right)}$ are zero, and other entries are placed in a lower triangular matrix. Moreover, most of these entries are zero due to the orthonormal property of the ODCPs. This structure makes the computational complexity of the methods based on this matrix to be greatly reduced. As a numerical example, in the case of m=5, we have

$$D_{5}^{\left(2\right)}=\left(\begin{array}{cccccc} 0 & 0 & 0 & 0 & 0 & 0\\ 0 & 0 & 0 & 0 & 0 & 0\\ \frac{75}{\sqrt{14}} & 0 & 0 & 0 & 0 & 0\\ 0 & \frac{125\;\sqrt{14}}{6} & 0 & 0 & 0 & 0\\ \frac{625\;\sqrt{42}}{28} & 0 & \frac{350\;\sqrt{3}}{3} & 0 & 0 & 0\\ 0 & \frac{2765\;\sqrt{10}}{12} & 0 & 90\;\sqrt{35} & 0 & 0 \end{array}\right)\text{.}$$

Theorem 2 Let 0<α⩽1 is a given number. The Caputo fractional derivative of order α of the vector $\Omega_{n}(t)$ defined in (3.6) satisfies the relation

(4.2) Dtα0cΩn(t)≃Qn(α)Ωn(t),

where $Q_{n}^{\left(\alpha \right)}=\left[q_{\mathrm{ij}}^{\left(\alpha \right)}\right]$ for 0⩽i,j⩽n and

$$q_{\mathrm{ij}}^{\left(\alpha \right)}=\left\{\begin{array}{cc} \sum_{l=0}^{n}\left(\sum_{k=1}^{i}\sum_{r=1}^{k}\frac{a_{n,i,k,r}\;r!}{\Gamma \left(r-\alpha +1\right)}{\left(\frac{l}{n}\right)}^{r-\alpha }\right)C_{n,j}\left(\frac{l}{n}\right), & \begin{array}{c} i=1,2,\ldots ,n,\\ j=0,1,\ldots n, \end{array}\\ 0, & \mathrm{otherwise}\text{.} \end{array}\right)$$

Notice that in the case of α=1, the index *j* in the above relation should be changed as j=0,1,…,i-1 (due to the orthonormal property of the ODCPs).

Proof From the formula expressed in (3.1) and the following property about the Caputo fractional derivative [1]:

Dtα0ctk=0,k=0,k!Γ(k-α+1)tk-α,k∈Z+,

we get

Dtα0cCn,i(t)=0,i=0,∑k=1i∑r=1kan,i,k,rr!Γ(r-α+1)tr-α,i=1,2,…,n.

Thus, from the above result and the definition of the vector $\Omega_{n}(t)$, we have

Dtα0cΩn(t)=0Dtα0cCn,1(t)Dtα0cCn,2(t)…Dtα0cCn,n(t)T.

The non-zero elements of the above vector can be approximated by the ODCPs as

Dtα0cCn,i(t)≃∑j=0nqij(α)Cn,j(t),

where

qij(α)=∑l=0nDtα0cCn,i(t)t=lnCn,jln.

The result obtained above, together with the formula expressed in (3.1), yield

$$q_{\mathrm{ij}}^{\left(\alpha \right)}=\overset{n}{\underset{l=0}{\sum }}\left(\overset{i}{\underset{k=1}{\sum }}\overset{k}{\underset{r=1}{\sum }}\frac{a_{n,i,k,r}\;r!}{\Gamma (r-\alpha +1)}{\left(\frac{l}{n}\right)}^{r-\alpha }\right)C_{n,j}\left(\frac{l}{n}\right)\text{.}$$

Thus, we get

Dtα0cΩn(t)≃00…00q10(α)q11(α)…q1n-1(α)q1n(α)q20(α)q21(α)…q2n-1(α)q2n(α)⋮⋮…⋮⋮qn0(α)qn1(α)…qnn-1(α)qnn(α)Ωn(t)≔Qn(α)Ωn(t).

Meanwhile, in the case of α=1, the orthonormal property of the ODCPs causes that $q_{\mathrm{ij}}^{\left(\alpha \right)}=0$ for j⩾i. Thus, the desired result is obtained.

As a numerical result, we get

$$Q_{5}^{\left(1/2\right)}=\left(\begin{array}{cccccc} 0.0 & 0.0 & 0.0 & 0.0 & 0.0 & 0.0\\ -2.06400282 & 1.04516634 & 0.28986745 & 0.13077572 & 0.05373220 & 0.01574645\\ -0.72577089 & -1.63871234 & 1.88160037 & 0.55311694 & 0.20781949 & 0.05884357\\ -1.15471519 & 0.66815186 & -1.71217641 & 2.38287740 & 0.57608349 & 0.14710398\\ 0.72673765 & -0.99783166 & 1.28860997 & -2.44140972 & 2.54613708 & 0.40193780\\ -0.85108059 & 3.28728970 & -2.89359550 & 1.63950839 & -4.28923103 & 2.55716711 \end{array}\right),$$

and

$$Q_{5}^{\left(1\right)}=\left(\begin{array}{cccccc} 0 & 0 & 0 & 0 & 0 & 0\\ -\frac{\sqrt{420}}{7} & 0 & 0 & 0 & 0 & 0\\ 0 & -\frac{-5\sqrt{1470}}{28} & 0 & 0 & 0 & 0\\ -\frac{37\;\sqrt{30}}{36} & 0 & -\frac{10\;\sqrt{105}}{9} & 0 & 0 & 0\\ 0 & -\frac{29\;\sqrt{490}}{42} & 0 & -3\;\sqrt{35} & 0 & 0\\ -\frac{461\;\sqrt{42}}{126} & 0 & -\frac{205\;\sqrt{147}}{63} & 0 & -30 & 0 \end{array}\right)\text{.}$$

Theorem 3 For the vector $\Omega_{n}(t)$ expressed in (3.6) and the given real number λ⩾0, the following relations are valid:

(4.3) $$e^{\lambda t}\Omega_{n}(t)≃H_{n}^{\left(\lambda \right)}\Omega_{n}(t),$$

and

(4.4) $$e^{-\lambda t}\Omega_{n}(t)≃K_{n}^{\left(\lambda \right)}\Omega_{n}(t),$$

where $H_{n}^{\left(\lambda \right)}=\left[h_{\mathrm{ij}}^{\left(\lambda \right)}\right]$ and $K_{n}^{\left(\lambda \right)}=\left[k_{\mathrm{ij}}^{\left(\lambda \right)}\right]$ for 0⩽i,j⩽n, such that

(4.5) $$h_{\mathrm{ij}}^{\left(\lambda \right)}=\overset{n}{\underset{l=0}{\sum }}\left(\overset{i}{\underset{k=0}{\sum }}\overset{k}{\underset{r=0}{\sum }}a_{n,i,k,r}e^{\lambda \frac{l}{n}}{\left(\frac{l}{n}\right)}^{r}\right)C_{n,j}\left(\frac{l}{n}\right),$$

and

(4.6) $$k_{\mathrm{ij}}^{\left(\lambda \right)}=\overset{n}{\underset{l=0}{\sum }}\left(\overset{i}{\underset{k=0}{\sum }}\overset{k}{\underset{r=0}{\sum }}a_{n,i,k,r}e^{-\lambda \frac{l}{n}}{\left(\frac{l}{n}\right)}^{r}\right)C_{n,j}\left(\frac{l}{n}\right)\text{.}$$

Proof From (3.1), we have

$$e^{\lambda t}C_{n,i}(t)=\overset{i}{\underset{k=0}{\sum }}\overset{k}{\underset{r=0}{\sum }}a_{n,i,k,r}e^{\lambda t}t^{r}$$

and

$$e^{\lambda t}\Omega_{n}(t)={\left[e^{\lambda t}C_{n,0}(t)\;\;e^{\lambda t}C_{n,1}(t)\ldots e^{\lambda t}C_{n,n}(t)\right]}^{T}\text{.}$$

The elements of the above vector can be approximated as

$$e^{\lambda t}C_{n,i}(t)≃\overset{n}{\underset{j=0}{\sum }}h_{\mathrm{ij}}^{\left(\lambda \right)}C_{n,j}(t),$$

where

$$h_{\mathrm{ij}}^{\left(\lambda \right)}=\overset{n}{\underset{l=0}{\sum }}{\left(\left(e^{\lambda t}C_{n,i}(t)\right)\right|}_{t=\frac{l}{n}}C_{n,j}\left(\frac{l}{n}\right)=\overset{n}{\underset{l=0}{\sum }}\left(\overset{i}{\underset{k=0}{\sum }}\overset{k}{\underset{r=0}{\sum }}a_{n,i,k,r}e^{\lambda \frac{l}{n}}{\left(\frac{l}{n}\right)}^{r}\right)C_{n,j}\left(\frac{l}{n}\right)\text{.}$$

Thus, from results obtained above, we have

$$e^{\lambda t}\Omega_{n}(t)≃\left(\begin{array}{ccccc} h_{00}^{\left(\lambda \right)} & h_{01}^{\left(\lambda \right)} & \ldots & h_{0n-1}^{\left(\lambda \right)} & h_{0n}^{\left(\lambda \right)}\\ h_{10}^{\left(\lambda \right)} & h_{11}^{\left(\lambda \right)} & \ldots & h_{1n-1}^{\left(\lambda \right)} & h_{1n}^{\left(\lambda \right)}\\ h_{20}^{\left(\lambda \right)} & h_{21}^{\left(\lambda \right)} & \ldots & h_{2n-1}^{\left(\lambda \right)} & h_{2n}^{\left(\lambda \right)}\\ \vdots & \vdots & \ldots & \vdots & \vdots \\ h_{n0}^{\left(\lambda \right)} & h_{n1}^{\left(\lambda \right)} & \ldots & h_{\mathrm{nn}-1}^{\left(\lambda \right)} & h_{\mathrm{nn}}^{\left(\lambda \right)} \end{array}\right)\Omega_{n}(t)≔H_{n}^{\left(\lambda \right)}\Omega_{n}(t)\text{.}$$

A similar argument can be made for the matrix $K_{n}^{\left(\lambda \right)}$. Thus, the proof is completed.

As a numerical example, by employing the formulas expressed in (4.5), (4.6), for λ=3/2 and n=5, we get

$$H_{5}^{\left(3/2\right)}=\left(\begin{array}{cccccc} 2.40556445 & -1.16896745 & 0.24995972 & -0.03245868 & 0.00271617 & -0.00013479\\ -1.16896745 & 2.61933900 & -1.02478258 & 0.19461828 & -0.02148295 & 0.00132535\\ 0.24995972 & -1.02478258 & 2.50261610 & -0.90298230 & 0.14773150 & -0.01209936\\ -0.03245868 & 0.19461828 & -0.90298230 & 2.41321437 & -0.75618756 & 0.09197494\\ 0.00271617 & -0.02148295 & 0.14773150 & -0.75618756 & 2.30851279 & -0.54506246\\ -0.00013479 & 0.00132535 & -0.01209936 & 0.09197494 & -0.54506246 & 2.18413997 \end{array}\right),$$

and

$$K_{5}^{\left(3/2\right)}=\left(\begin{array}{cccccc} 0.53675398 & 0.26083189 & 0.05577355 & 0.00724251 & 0.00060606 & 0.00003007\\ 0.26083189 & 0.58445353 & 0.22865990 & 0.04342521 & 0.00479349 & 0.00029572\\ 0.05577355 & 0.22865990 & 0.55840913 & 0.20148258 & 0.03296335 & 0.00269973\\ 0.00724251 & 0.04342521 & 0.20148258 & 0.53846091 & 0.16872825 & 0.02052238\\ 0.00060606 & 0.00479349 & 0.03296335 & 0.16872825 & 0.51509882 & 0.12161987\\ 0.00003007 & 0.00029572 & 0.00269973 & 0.02052238 & 0.12161987 & 0.48734750 \end{array}\right)\text{.}$$

Theorem 4 Let 0<α⩽1 and λ⩾0 are given real numbers, and $\Omega_{n}(t)$ is the vector defined in (3.6). The tempered fractional derivative of $\Omega_{n}(t)$ can be computed as

(4.7) Dtα,λ0cΩn(t)≃Pn(α,λ)Ωn(t),

where $P_{n}^{\left(\alpha ,\lambda \right)}$ is an (n+1)-order matrix that can be calculated as $P_{n}^{\left(\alpha ,\lambda \right)}=H_{n}^{\left(\lambda \right)}Q_{n}^{\left(\alpha \right)}K_{n}^{\left(\lambda \right)}$ such that the operational matrices $Q_{n}^{\left(\alpha \right)},H_{n}^{\left(\lambda \right)}$ and $K_{n}^{\left(\lambda \right)}$ are already introduced in Theorem 2, Theorem 3.

Proof From the definition of the tempered fractional derivative, we have

Dtα,λ0cΩn(t)=e-λtDtα0ceλtΩn(t),

which by employing Theorem 3, we get

Dtα,λ0cΩn(t)≃Hn(λ)e-λtDtα0cΩn(t),

and, subsequently by applying Theorem 2, we obtain

Dtα,λ0cΩn(t)≃Hn(λ)Qn(α)e-λtΩn(t).

Eventually, from the result obtained above, and Theorem 3, we have

Dtα,λ0cΩn(t)≃Hn(λ)Qn(α)Kn(λ)Ωn(t)≔Pn(α,λ)Ωn(t),

which completes the proof.

As a numerical example, we get

$$P_{5}^{\left(1/2,3/2\right)}=\left(\begin{array}{cccccc} 0.79684463 & -0.33639527 & -0.16608677 & -0.07409196 & -0.02924888 & -0.00838339\\ -1.52295175 & 1.05543478 & -0.02574639 & 0.00903072 & 0.00798324 & 0.00271596\\ -0.43622388 & -1.74429150 & 1.92485058 & 0.37063473 & 0.15513814 & 0.04704186\\ -0.90052793 & 0.42591864 & -1.66872637 & 2.48720597 & 0.47351608 & 0.11200612\\ 0.60165812 & -0.99553285 & 1.06190639 & -2.09483624 & 2.74123617 & 0.51839426\\ 0.40112246 & 2.47413700 & -1.67405114 & -0.06590093 & -4.19702934 & 1.40737617 \end{array}\right)\text{.}$$

## The established methods

In this section, the ODCPs along with the results obtained for them in the previous section are used to solve the problems given in (1), (2). The developed methods are based on the expression of the unknown solution of the problem under investigation by a finite series of the ODCPs with some unknown coefficients and finding these coefficients.

### The established method for the first problem

To obtain a unique solution for the tempered time fractional equation expressed in (1), we first accompany this equation with the following conditions:

(5.1) $${\left(\Psi \right|}_{t=0}=\vartheta ,$$

and

(5.2) $$\begin{array}{cc} {\left(\Psi \right|}_{x=0}=\mu , & {\left(\Psi \right|}_{x=1}=\nu , \end{array}$$

where ϑ=ϑ(x),μ=μ(t) and ν=ν(t) are given complex functions. The known and unknown complex functions in this problem can be represented as

(5.3) $$\begin{array}{ccc} \Psi =\Psi_{1}+i\;\Psi_{2}, & f=f_{1}+i\;f_{2}, & \vartheta =\vartheta_{1}+i\;\vartheta_{2},\\ \mu =\mu_{1}+i\;\mu_{2}, & \nu =\nu_{1}+i\;\nu_{2}, & \; \end{array}$$

where $\Psi_{1}=\Psi_{1}(x,t)$ and $\Psi_{2}=\Psi_{2}(x,t)$ are unknown real functions, $f_{1}=f_{1}\left(x,t\right),\;f_{2}=f_{2}\left(x,t\right),\vartheta_{1}=\vartheta_{1}\left(x\right),\vartheta_{2}=\vartheta_{2}\left(x\right),\mu_{1}=\mu_{1}\left(t\right),\mu_{2}=\mu_{2}\left(t\right),\nu_{1}=\nu_{1}\left(t\right)$ and $\nu_{2}=\nu_{2}(t)$ are known real functions. Based on the representation above, solving the main tempered time fractional problem (1) under the conditions given in (5.1), (5.2) turns into solving the following system of tempered time fractional equations:

(5.4) -Dtα,λ0cΨ2+θ∂xxΨ1+σΨ13+Ψ1Ψ22+ηΨ1=f1,Dtα,λ0cΨ1+θ∂xxΨ2+σΨ12Ψ2+Ψ23+ηΨ2=f2,

under the conditions

(5.5) $$\begin{array}{cc} {\left(\Psi_{1}\right|}_{t=0}=\vartheta_{1}, & {\left(\Psi_{2}\right|}_{t=0}=\vartheta_{2}, \end{array}$$

and

(5.6) $$\begin{array}{cc} {\left(\Psi_{1}\right|}_{x=0}=\mu_{1}, & {\left(\Psi_{2}\right|}_{x=0}=\mu_{2},\\ {\left(\Psi_{1}\right|}_{x=1}=\nu_{1}, & {\left(\Psi_{2}\right|}_{x=1}=\nu_{2}\text{.} \end{array}$$

To solve the system introduced in (5.4), (5.6), we approximate the unknown functions as

(5.7) $$\begin{array}{c} \Psi_{1}≃\overset{m}{\underset{i=0}{\sum }}\overset{n}{\underset{j=0}{\sum }}w_{\mathrm{ij}}C_{m,i}(x)C_{n,j}(t)≔\Omega_{m}^{T}(x)W_{m\times n}\Omega_{n}(t),\\ \Psi_{2}≃\overset{m}{\underset{i=0}{\sum }}\overset{n}{\underset{j=0}{\sum }}z_{\mathrm{ij}}C_{m,i}(x)C_{n,j}(t)≔\Omega_{m}^{T}(x)Z_{m\times n}\Omega_{n}(t), \end{array}$$

where $W_{m\times n}=\left[w_{\mathrm{ij}}\right]$ and $Z_{m\times n}=\left[z_{\mathrm{ij}}\right]$ for 0⩽i⩽m and 0⩽j⩽n are undetermined matrices. From relation (5.7) and Theorem 4, we get

(5.8) Dtα,λ0cΨ1≃ΩmT(x)Wm×nPn(α,λ)Ωn(t),Dtα,λ0cΨ2≃ΩmT(x)Zm×nPn(α,λ)Ωn(t).

Also, relation (5.7), along with Theorem 1, result in

(5.9) $$\begin{array}{c} \partial_{\mathrm{xx}}\Psi_{1}≃\Omega_{m}^{T}(x){\left(D_{m}^{\left(2\right)}\right)}^{T}W_{m\times n}\Omega_{n}(t),\\ \partial_{\mathrm{xx}}\Psi_{2}≃\Omega_{m}^{T}(x){\left(D_{m}^{\left(2\right)}\right)}^{T}Z_{m\times n}\Omega_{n}(t)\text{.} \end{array}$$

The following residual functions can be defined by substituting (5.7), (5.9) into (5.4):

(5.10) $$\begin{array}{c} R_{1}\left(x,t\right)≔\Omega_{m}^{T}\left(x\right)\left[\eta \left(x\right)W_{m\times n}-Z_{m\times n}P_{n}^{\left(\alpha ,\lambda \right)}+\theta {\left(D_{m}^{\left(2\right)}\right)}^{T}W_{m\times n}\right]\Omega_{n}\left(t\right)-f_{1}\left(x,t\right)\\ +\sigma {\left(\Omega_{m}^{T}\left(x\right)W_{m\times n}\Omega_{n}\left(t\right)\right)}^{3}+\sigma \left(\Omega_{m}^{T}\left(x\right)W_{m\times n}\Omega_{n}\left(t\right)\right){\left(\Omega_{m}^{T}\left(x\right)Z_{m\times n}\Omega_{n}\left(t\right)\right)}^{2}≃0,\\ R_{2}\left(x,t\right)≔\Omega_{m}^{T}\left(x\right)\left[W_{m\times n}P_{n}^{\left(\alpha ,\lambda \right)}+\theta {\left(D_{m}^{\left(2\right)}\right)}^{T}Z_{m\times n}+\eta \left(x\right)Z_{m\times n}\right]\Omega_{n}\left(t\right)-f_{2}\left(x,t\right)\\ +\sigma {\left(\Omega_{m}^{T}\left(x\right)W_{m\times n}\Omega_{n}\left(t\right)\right)}^{2}\left(\Omega_{m}^{T}\left(x\right)Z_{m\times n}\Omega_{n}\left(t\right)\right)+\sigma {\left(\Omega_{m}^{T}\left(x\right)Z_{m\times n}\Omega_{n}\left(t\right)\right)}^{3}≃0\text{.} \end{array}$$

Meanwhile, we derive the following results from (5.5), (5.7):

(5.11) $$\begin{array}{cc} {\tilde{\vartheta }}_{1}\left(x\right)≔\Omega_{m}^{T}\left(x\right)W_{m\times n}\Omega_{n}\left(0\right)-\vartheta_{1}\left(x\right)≃0, & \end{array}\;{\tilde{\vartheta }}_{2}\left(x\right)≔\Omega_{m}^{T}\left(x\right)Z_{m\times n}\Omega_{n}\left(0\right)-\vartheta_{2}\left(x\right)≃0$$

and

(5.12) $$\begin{array}{cc} {\tilde{\mu }}_{1}\left(t\right)≔\Omega_{m}^{T}\left(0\right)W_{m\times n}\Omega_{n}\left(t\right)-\mu_{1}\left(t\right)≃0, & \\ {\tilde{\mu }}_{2}\left(t\right)≔\Omega_{m}^{T}\left(0\right)Z_{m\times n}\Omega_{n}\left(t\right)-\mu_{2}\left(t\right)≃0, & \\ {\tilde{\nu }}_{1}\left(t\right)≔\Omega_{m}^{T}\left(1\right)W_{m\times n}\Omega_{n}\left(t\right)-\nu_{1}\left(t\right)≃0, & \\ {\tilde{\nu }}_{2}\left(t\right)≔\Omega_{m}^{T}\left(1\right)Z_{m\times n}\Omega_{n}\left(t\right)-\nu_{2}\left(t\right)≃0\text{.} & \end{array}$$

Now from (5.10), (5.12), we build the following system of 2(m+1)(n+1) nonlinear algebraic equations:

(5.13) $$\begin{array}{cc} R_{k}\left(\frac{i}{m},\frac{j}{n}\right)=0, & 1⩽i⩽m-1,\;\;1⩽j⩽n,\\ {\overset{∼}{\vartheta }}_{k}\left(\frac{i}{m}\right)=0, & 1⩽i⩽m-1,\\ {\overset{∼}{\mu }}_{k}\left(\frac{j}{n}\right)=0, & 0⩽j⩽n,\\ {\overset{∼}{\nu }}_{k}\left(\frac{j}{n}\right)=0, & 0⩽j⩽n, \end{array}$$

where k=1,2. Eventually, by finding a numerical solution for the algebraic system derived above (determining the elements of the matrices $W_{m\times n}$ and $Z_{m\times n}$) with specific values α and λ, a numerical solution is derived for the main tempered time fractional system (5.4) (or equivalently for the tempered time fractional Eq. (1)) with the aid of the approximations considered in (5.7). The ”fsolve” command in Maple 18 (with 25 decimal digits) has been employed to find a numerical solution for the algebraic system (5.13). It is also used for other numerical purposes in the continuation. The step-by-step algorithm of the proposed method is given as follows:

**Table:** 

**Algorithm of the proposed method for the first problem**
Input: The numbers α,λ,θ,σ and η. The functions $f_{l},\vartheta_{l},\mu_{l}$ and $\nu_{l}$ for l=1,2.
> Define the functions $C_{m,i}(x),0⩽i⩽m$ and $C_{n,j}(t),0⩽j⩽n$ using (3.1).
> Introduce the vectors $\Omega_{m}(x)$ and $\Omega_{n}(t)$ like (3.5).
> Calculate the matrices $D_{m}^{\left(2\right)}$ and $P_{n}^{\left(\alpha ,\lambda \right)}$ using Theorem 1, Theorem 4, respectively.
> Define the (m+1)×(n+1) matrices $W_{m\times n}=\left[w_{\mathrm{ij}}\right]$ and $Z_{m\times n}=\left[z_{\mathrm{ij}}\right]$ like (5.7).
> Calculate the approximations given in (5.8), (5.9).
> Define the residual functions given in (5.10).
> Define the approximations given in (5.11), (5.12).
> Extract the algebraic system (5.13) and solve it.
> Assign the coefficients matrices $W_{m\times n}$ and $Z_{m\times n}$.
Output: Numerical solution $\left\{\Psi_{1}(x,t)≃\Omega_{m}^{T}(x)W_{m\times n}\Omega_{n}(t),\;\Psi_{2}(x,t)≃\Omega_{m}^{T}(x)Z_{m\times n}\Omega_{n}(t)\right\}$.

### The established method for the second problem

Here we establish a collocation method based on the ODCPs to solve the tempered time fractional problem (1) under the following conditions:

(5.14) Ψt=0=ϑ^,Φt=0=ϑ¯,

and

(5.15) Ψx=0=μ^,Ψx=1=ν^,Φx=0=μ¯,Φx=1=ν¯,

where ϑ^=ϑ^(x),ϑ¯=ϑ¯(x),μ^=μ^(t),ν^=ν^(t),μ¯=μ¯(t) and $\overset{¯}{\nu }=\overset{¯}{\nu }(t)$ are given complex functions. The complex functions (known and unknown) in this system can be rewritten as

(5.16) $$\begin{array}{ccccc} \Psi ={\hat{\Psi }}_{1}+i\;{\hat{\Psi }}_{2}, & g=g_{1}+i\;g_{2}, & \hat{\vartheta }={\hat{\vartheta }}_{1}+i\;{\hat{\vartheta }}_{2}, & \hat{\mu }={\hat{\mu }}_{1}+i\;{\hat{\mu }}_{2}, & \\ \hat{\nu }={\hat{\nu }}_{1}+i\;{\hat{\nu }}_{2}, & \Phi ={\bar{\Phi }}_{1}+i\;{\bar{\Phi }}_{2}, & h=h_{1}+i\;h_{2}, & \bar{\vartheta }={\bar{\vartheta }}_{1}+i\;{\bar{\vartheta }}_{2}, & \\ \bar{\mu }={\bar{\mu }}_{1}+i\;{\bar{\mu }}_{2}, & \bar{\nu }={\bar{\nu }}_{1}+i\;{\bar{\nu }}_{2}, & & & \end{array}$$

where ${\hat{\Psi }}_{1}={\hat{\Psi }}_{1}\left(x,t\right),\;{\hat{\Psi }}_{2}={\hat{\Psi }}_{2}\left(x,t\right),{\bar{\;\Phi }}_{1}={\bar{\Phi }}_{1}\left(x,t\right)$ and ${\overset{¯}{\Phi }}_{2}={\overset{¯}{\Phi }}_{2}(x,t)$ are unknown real functions, $g_{1}=g_{1}\left(x,t\right),g_{2}=g_{2}\left(x,t\right),h_{1}=h_{1}\left(x,t\right),\;h_{2}=h_{2}\left(x,t\right),{\hat{\;\vartheta }}_{1}={\hat{\vartheta }}_{1}\left(x\right),\;{\hat{\vartheta }}_{2}={\hat{\vartheta }}_{2}\left(x\right),{\hat{\;\mu }}_{1}={\hat{\mu }}_{1}\left(t\right),{\hat{\;\mu }}_{2}={\hat{\mu }}_{2}\left(t\right),{\hat{\;\nu }}_{1}={\hat{\nu }}_{1}\left(t\right),{\hat{\;\nu }}_{2}={\hat{\nu }}_{2}\left(t\right),{\bar{\;\vartheta }}_{1}={\bar{\vartheta }}_{1}\left(x\right),{\bar{\;\vartheta }}_{2}={\bar{\vartheta }}_{2}\left(x\right),{\bar{\;\mu }}_{1}={\bar{\mu }}_{1}\left(t\right),{\bar{\;\mu }}_{2}={\bar{\mu }}_{2}\left(t\right),{\overset{\;¯}{\nu }}_{1}={\bar{\nu }}_{1}\left(t\right)$ and ${\overset{¯}{\nu }}_{2}={\overset{¯}{\nu }}_{2}(t)$ are known real functions. Thus, solving the main tempered time fractional system (2) with the conditions given in (5.14), (5.15) transforms into solving the following equivalent system of tempered time fractional equations:

(5.17) -Dtα,λ0cΨ^2+θ∂xxΨ^1+σ1Ψ^12+Ψ^22+σ2Φ¯12+Φ¯22Ψ^1+η1Ψ^1+η2Φ¯1=g1,Dtα,λ0cΨ^1+θ∂xxΨ^2+σ1Ψ^12+Ψ^22+σ2Φ¯12+Φ¯22Ψ^2+η1Ψ^2+η2Φ¯2=g2,-Dtα,λ0cΦ¯2+θ∂xxΦ¯1+σ1Ψ^12+Ψ^22+σ2Φ¯12+Φ¯22Φ¯1+η1Φ¯1+η2Ψ^1=h1,Dtα,λ0cΦ¯1+θ∂xxΦ¯2+σ1Ψ^12+Ψ^22+σ2Φ¯12+Φ¯22Φ¯2+η1Φ¯2+η2Ψ^2=h2,

under the conditions

(5.18) Ψ^1t=0=ϑ^1,Ψ^2t=0=ϑ^2,Φ¯1t=0=ϑ¯1,Φ¯2t=0=ϑ¯2,

and

(5.19) Ψ^1x=0=μ^1,Ψ^2x=0=μ^2,Ψ^1x=1=ν^1,Ψ^2x=1=ν^2,Φ¯1x=0=μ¯1,Φ¯2x=0=μ¯2,Φ¯1x=1=ν¯1,Ψ¯2x=1=ν¯2.

To solve the problem expressed in (5.17), (5.19), we approximate the unknown functions by the ODCPs as

(5.20) Ψ^1≃∑i=0m∑j=0nw^ijCm,i(x)Cn,j(t)≔ΩmT(x)W^m×nΩn(t),Ψ^2≃∑i=0m∑j=0nz^ijCm,i(x)Cn,j(t)≔ΩmT(x)Z^m×nΩn(t),Φ¯1≃∑i=0m∑j=0nw¯ijCm,i(x)Cn,j(t)≔ΩmT(x)W‾m×nΩn(t),Φ¯2≃∑i=0m∑j=0nz¯ijCm,i(x)Cn,j(t)≔ΩmT(x)Z‾m×nΩn(t),

where W^m×n=w^ij,Z^m×n=z^ij,W‾m×n=w¯ij and ${\overset{‾}{Z}}_{m\times n}=\left[{\overset{¯}{z}}_{\mathrm{ij}}\right]$ for 0⩽i⩽m and 0⩽j⩽n are undetermined matrices. Theorem 4, together with relation (5.20) result in

(5.21) Dtα,λ0cΨ^1≃ΩmT(x)W^m×nPn(α,λ)Ωn(t),Dtα,λ0cΨ^2≃ΩmT(x)Z^m×nPn(α,λ)Ωn(t),Dtα,λ0cΦ¯1≃ΩmT(x)W‾m×nPn(α,λ)Ωn(t),Dtα,λ0cΦ¯2≃ΩmT(x)Z‾m×nPn(α,λ)Ωn(t).

From Theorem 1 and relation (5.20), we have

(5.22) ∂xxΨ^1≃ΩmT(x)Dm(2)TW^m×nΩn(t),∂xxΨ^2≃ΩmT(x)Dm(2)TZ^m×nΩn(t),∂xxΦ¯1≃ΩmT(x)Dm(2)TW‾m×nΩn(t),∂xxΦ¯2≃ΩmT(x)Dm(2)TZ‾m×nΩn(t).

By substituting (5.20)–(5.22) into (5.17), we get

(5.23) R^1(x,t)≔ΩmT(x)-Z^m×nPn(α,λ)+θDm(2)TW^m×n+η1(x)W^m×n+η2(x)W‾m×nΩn(t)+σ1ΩmT(x)W^m×nΩn(t)2+ΩmT(x)Z^m×nΩn(t)2+σ2ΩmT(x)W‾m×nΩn(t)2+ΩmT(x)Z‾m×nΩn(t)2ΩmT(x)W^m×nΩn(t)-g1(x,t)≃0,R^2(x,t)≔ΩmT(x)W^m×nPn(α,λ)+θDm(2)TZ^m×n+η1(x)Z^m×n+η2(x)Z‾m×nΩn(t)+σ1ΩmT(x)W^m×nΩn(t)2+ΩmT(x)Z^m×nΩn(t)2+σ2ΩmT(x)W‾m×nΩn(t)2+ΩmT(x)Z‾m×nΩn(t)2ΩmT(x)Z^m×nΩn(t)-g2(x,t)≃0,R‾1(x,t)≔ΩmT(x)-Z‾m×nPn(α,λ)+θDm(2)TW‾m×n+η1(x)W‾m×n+η2(x)W^m×nΩn(t)+σ1ΩmT(x)W^m×nΩn(t)2+ΩmT(x)Z^m×nΩn(t)2+σ2ΩmT(x)W‾m×nΩn(t)2+ΩmT(x)Z‾m×nΩn(t)2ΩmT(x)W‾m×nΩn(t)-h1(x,t)≃0,R‾2(x,t)≔ΩmT(x)W‾m×nPn(α,λ)+θDm(2)TZ‾m×n+η1(x)Z‾m×n+η2(x)Z^m×nΩn(t)+σ1ΩmT(x)W^m×nΩn(t)2+ΩmT(x)Z^m×nΩn(t)2+σ2ΩmT(x)W‾m×nΩn(t)2+ΩmT(x)Z‾m×nΩn(t)2ΩmT(x)Z‾m×nΩn(t)-h2(x,t)≃0,

Also, from (5.18)–(5.20), we get

(5.24) ϑ^1(x)≔ΩmT(x)W^m×nΩn(0)-ϑ^1(x)≃0,ϑ^2(x)≔ΩmT(x)Z^m×nΩn(0)-ϑ^2(x)≃0,ϑ‾1(x)≔ΩmT(x)W‾m×nΩn(0)-ϑ¯1(x)≃0,ϑ‾2(x)≔ΩmT(x)Z‾m×nΩn(0)-ϑ¯2(x)≃0,

and

(5.25) μ^1(t)≔ΩmT(0)W^m×nΩn(t)-μ^1(t)≃0,μ^2(t)≔ΩmT(0)Z^m×nΩn(t)-μ^2(t)≃0,ν^1(t)≔ΩmT(1)W^m×nΩn(t)-ν^1(t)≃0,ν^2(t)≔ΩmT(1)Z^m×nΩn(t)-ν^2(t)≃0,μ‾1(t)≔ΩmT(0)W‾m×nΩn(t)-μ¯1(t)≃0,μ‾2(t)≔ΩmT(0)Z‾m×nΩn(t)-μ¯2(t)≃0,ν‾1(t)≔ΩmT(1)W‾m×nΩn(t)-ν¯1(t)≃0,ν‾2(t)≔ΩmT(1)Z‾m×nΩn(t)-ν¯2(t)≃0.

From (5.23), (5.25), we extract the nonlinear system of equations

(5.26) R^kim,jn=0,R‾kim,jn=0,1⩽i⩽m-1,1⩽j⩽n,ϑ^kim=0,ϑ‾kim=0,1⩽i⩽m-1,μ^kjn=0,μ‾kjn=0,0⩽j⩽n,ν^kjn=0,ν‾kjn=0,0⩽j⩽n,

where k=1,2. Finally, by finding a numerical solution for the above system (determining the entries of the matrices W^m×n,Z^m×n,W‾m×n and ${\overset{‾}{Z}}_{m\times n}$) with specific values α and λ, a numerical solution is obtained for the system (5.17) (or equivalently for the tempered time fractional system (2)) through the approximations expressed in (5.20). The step-by-step algorithm of the established method is given in the continuation.

**Table:** 

**Algorithm of the proposed method for the second problem**
Input: The numbers $\alpha ,\lambda ,\theta ,\sigma_{1},\sigma_{2},\eta_{1}$ and $\eta_{2}$. The functions gl,hl,ϑ^l,ϑ¯l,μ^l,μ¯l,ν^l and ${\overset{¯}{\nu }}_{l}$ for l=1,2.
> Define the functions $C_{m,i}(x),0⩽i⩽m$ and $C_{n,j}(t),0⩽j⩽n$ using (3.1).
> Introduce the vectors $\Omega_{m}(x)$ and $\Omega_{n}(t)$ like (3.5).
> Calculate the matrices $D_{m}^{\left(2\right)}$ and $P_{n}^{\left(\alpha ,\lambda \right)}$ using Theorem 1, Theorem 4, respectively.
> Define the (m+1)×(n+1) matrices W^m×n=w^ij,Z^m×n=z^ij,W‾m×n=w¯ij and ${\overset{‾}{Z}}_{m\times n}=\left[{\overset{¯}{z}}_{\mathrm{ij}}\right]$ like (5.20).
> Calculate the approximations given in (5.21), (5.22).
> Define the residual functions given in (5.23).
> Define the approximations given in (5.24), (5.25).
> Extract the algebraic system (5.26) and solve it.
> Assign the coefficients matrices W^m×n,Z^m×n,W‾m×n and ${\overset{‾}{Z}}_{m\times n}$.
Output: Numerical solution
Ψ1^(x,t)≃ΩmT(x)W^m×nΩn(t),Ψ^2(x,t)≃ΩmT(x)Z^m×nΩn(t),Φ¯1≃ΩmT(x)W‾m×nΩn(t),Φ¯2≃ΩmT(x)Z‾m×nΩn(t).

## Numerical examples

In order to acknowledge the high accuracy of the described methods, we investigate some examples. The following formulas are used in this regard:

$$\begin{array}{c} e_{\Psi_{1}}={\left(\int_{0}^{1}\int_{0}^{1}{\left(\Psi_{1}(x,t)-{\overset{\sim }{\Psi }}_{1}(x,t)\right)}^{2}\mathrm{dxdt}\right)}^{1/2},\\ e_{\Psi_{2}}={\left(\int_{0}^{1}\int_{0}^{1}{\left(\Psi_{2}(x,t)-{\overset{\sim }{\Psi }}_{2}(x,t)\right)}^{2}\mathrm{dxdt}\right)}^{1/2}, \end{array}$$

where $\Psi_{1}(x,t)$ and $\Psi_{2}(x,t)$ are the exact solutions, and ${\overset{\sim }{\Psi }}_{1}(x,t)$ and ${\overset{\sim }{\Psi }}_{2}(x,t)$ are the numerical solutions. All numerical computations are performed on a X64-based PC with Intel (R) Core (TM) i7-7500U CPU @ 2.90 GHz and 32.0 GB of RAM.

Example 1 Consider the equation given in (1) with $\theta =\sigma =1,\eta (x)=\sin^{2}(x)$ and

$$\begin{array}{c} f(x,t)=-\frac{6}{\Gamma (4-\alpha )}t^{3-\alpha }e^{-\lambda t}\sin(x)+t^{3}e^{-\lambda \;t}\cos\left(x\right)\left(t^{6}e^{-2\lambda t}-\cos^{2}\left(x\right)\right)\\ +i\left(\frac{6}{\Gamma (4-\alpha )}t^{3-\alpha }e^{-\lambda t}\cos(x)+t^{3}e^{-\lambda t}\sin(x)\left(t^{6}e^{-2\lambda t}-\cos^{2}\left(x\right)\right)\right), \end{array}$$

where

$${\left(\Psi \right|}_{t=0}=0,$$

and

$$\begin{array}{cc} {\left(\Psi \right|}_{x=0}=t^{3}e^{-\lambda t}, & {\left(\Psi \right|}_{x=1}=t^{3}e^{-\lambda t+i}\text{.} \end{array}$$

**Fig. 1:**
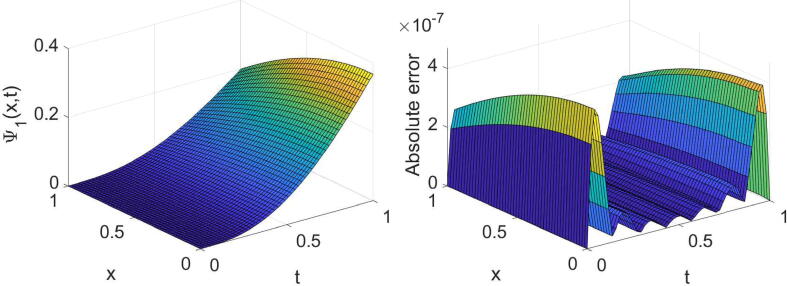
Plots of the obtained solution $\Psi_{1}$ and associated error with α=1/4,λ=1 and (m=n=7) in Exa.mple 1.

**Fig. 2:**
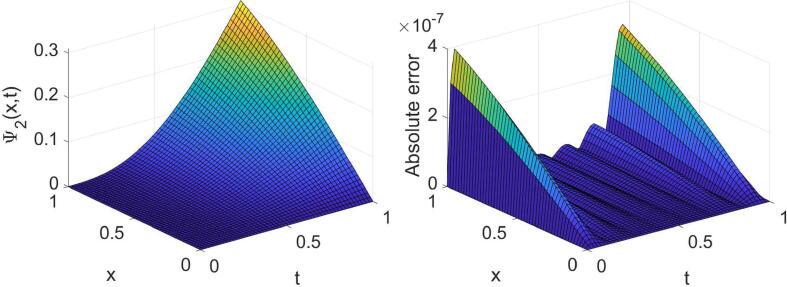
Plots of the obtained solution $\Psi_{2}$ and associated error with α=1/4,λ=1 and (m=n=7) in Exa.mple 1.

The equation’s exact solution is $\Psi (x,t)=t^{3}e^{-\lambda t+\mathrm{ix}}$. The results extracted of the established technique for three values of α, two values of λ and some values of (m,n) are reported in Table 1. The high accuracy and high convergence of the results can easily be observed from this table. For the specific case, α=1/4,λ=1 and (m=n=7), the obtained results are shown graphically in Fig. 1, Fig. 2.

**Table 1:** The errors obtained by the established algorithm with some values of (m,n) in Example 1 for some choices of α and λ.

			α					
			1/4		1/2		3/4	
λ	*m*	*n*	$e_{\Psi_{1}}$	$e_{\Psi_{2}}$	$e_{\Psi_{1}}$	$e_{\Psi_{2}}$	$e_{\Psi_{1}}$	$e_{\Psi_{2}}$
1	4	4	$2.8825\times 10^{-4}$	$1.7736\times 10^{-4}$	$2.8827\times 10^{-4}$	$1.7734\times 10^{-4}$	$2.8829\times 10^{-4}$	$1.7731\times 10^{-4}$
	5	5	$2.9549\times 10^{-5}$	$1.8108\times 10^{-5}$	$2.9540\times 10^{-5}$	$1.8116\times 10^{-5}$	$2.9527\times 10^{-5}$	$1.8126\times 10^{-5}$
	6	6	$2.2314\times 10^{-6}$	$1.3635\times 10^{-6}$	$2.2314\times 10^{-6}$	$1.3636\times 10^{-6}$	$2.2313\times 10^{-6}$	$1.3637\times 10^{-6}$
	7	7	$1.3760\times 10^{-7}$	$8.4226\times 10^{-8}$	$1.3762\times 10^{-7}$	$8.4208\times 10^{-8}$	$1.3765\times 10^{-7}$	$8.4187\times 10^{-8}$
3	4	4	$2.6732\times 10^{-4}$	$1.6370\times 10^{-4}$	$2.6732\times 10^{-4}$	$1.6370\times 10^{-4}$	$2.6732\times 10^{-4}$	$1.6370\times 10^{-4}$
	5	5	$1.3185\times 10^{-4}$	$8.0734\times 10^{-5}$	$1.3185\times 10^{-4}$	$8.0734\times 10^{-5}$	$1.3185\times 10^{-4}$	$8.0735\times 10^{-5}$
	6	6	$3.7016\times 10^{-5}$	$2.2664\times 10^{-5}$	$3.7016\times 10^{-5}$	$2.2664\times 10^{-5}$	$3.7016\times 10^{-5}$	$2.2664\times 10^{-5}$
	7	7	$7.7270\times 10^{-6}$	$4.7312\times 10^{-6}$	$7.7270\times 10^{-6}$	$4.7312\times 10^{-6}$	$7.7270\times 10^{-6}$	$4.7312\times 10^{-6}$

Example 2 Consider the tempered time fractional equation expressed in (1) with $\theta =\sigma =2,\eta (x)=e^{-x}$ and

$$\begin{array}{c} f(x,t)=\cos(2x)t^{1-\alpha }e^{-\lambda t}E_{1,2-\alpha }(-t)+e^{-(\lambda +1)t}\sin(2x)\left(2e^{-(\lambda +1)t}+e^{-x}-8\right)\\ +i\left(-\sin(2x)t^{1-\alpha }e^{-\lambda t}E_{1,2-\alpha }(-t)+e^{-(\lambda +1)t}\cos(2x)\left(2e^{-(\lambda +1)t}+e^{-x}-8\right)\right), \end{array}$$

where

$${\left(\Psi \right|}_{t=0}=\sin(2x)+i\cos(2x),$$

and

$$\begin{array}{cc} {\left(\Psi \right|}_{x=0}={\mathrm{ie}}^{-(\lambda +1)t}, & {\left(\Psi \right|}_{x=1}=e^{-(\lambda +1)}\left(\sin(2)+i\cos(2)\right)\text{.} \end{array}$$

The problem’s analytic solution is $\Psi (x,t)=e^{-(\lambda +1)t}\left(\sin(2x)+i\cos(2x)\right)$. Notice that the function $E_{1,2-\alpha }(-t)$ expressed in this problem is the Mittag–Leffler function. We remind that the Mittag–Leffler function with two positive parameters $\xi_{1}$ and $\xi_{2}$ is defined as [1]

$$E_{\xi_{1},\xi_{2}}(t)=\overset{\infty }{\underset{k=0}{\sum }}\frac{t^{k}}{\Gamma (k\xi_{1}+\xi_{2})}\text{.}$$

**Fig. 3:**
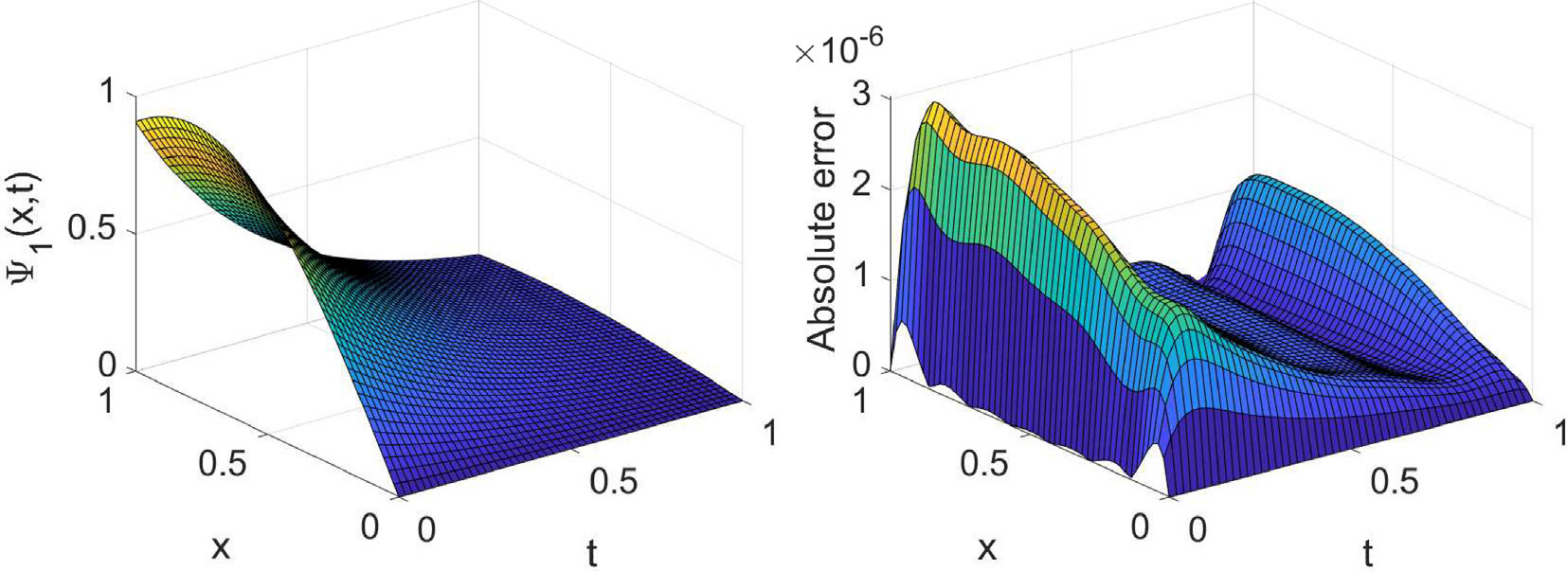
Plots of the obtained solution $\Psi_{1}$ and associated error with α=1/2,λ=3/2 and (m=n=7) in Exa.mple 2.

**Fig. 4:**
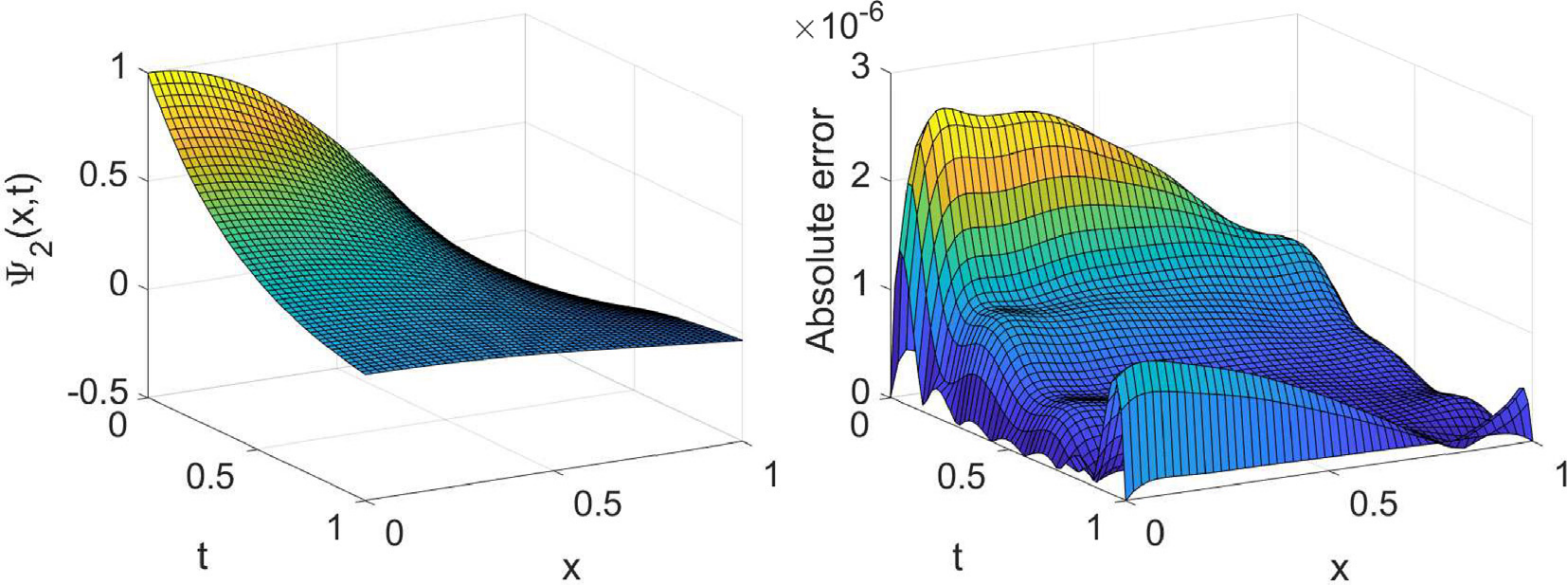
Plots of the obtained solution $\Psi_{2}$ and associated error with α=1/2,λ=3/2 and (m=n=7) in Exa.mple 2.

In this study, we used an approximation of the above infinite series with the first 25 terms for the numerical purposes. In Table 2, the results extracted by the technique proposed for such a problem are displayed for three values of α, two values of λ and some values of (m,n). The high accuracy and high convergence of the outcomes can easily be concluded from this table. In the case of α=1/2,λ=3/2 and (m=n=7), the extracted results are displayed graphically in Fig. 3, Fig. 4.

**Table 2:** The errors obtained by the established algorithm with some values of (m,n) in Example 2 for some choices of α and λ.

			α					
			1/4		1/2		3/4	
λ	*m*	*n*	$e_{\Psi_{1}}$	$e_{\Psi_{2}}$	$e_{\Psi_{1}}$	$e_{\Psi_{2}}$	$e_{\Psi_{1}}$	$e_{\Psi_{2}}$
1/4	4	4	$4.2157\times 10^{-4}$	$5.6267\times 10^{-4}$	$4.2179\times 10^{-4}$	$5.6295\times 10^{-4}$	$4.2240\times 10^{-4}$	$5.6383\times 10^{-4}$
	5	5	$1.3777\times 10^{-4}$	$1.0053\times 10^{-4}$	$1.3857\times 10^{-4}$	$1.0033\times 10^{-4}$	$1.4209\times 10^{-4}$	$9.8131\times 10^{-5}$
	6	6	$5.4721\times 10^{-6}$	$7.7576\times 10^{-6}$	$5.4725\times 10^{-6}$	$7.7613\times 10^{-6}$	$5.4649\times 10^{-6}$	$7.7793\times 10^{-6}$
	7	7	$1.4651\times 10^{-6}$	$1.0680\times 10^{-6}$	$1.4727\times 10^{-6}$	$1.0690\times 10^{-6}$	$1.5169\times 10^{-6}$	$1.0492\times 10^{-6}$
3/2	4	4	$4.5295\times 10^{-4}$	$5.0772\times 10^{-4}$	$4.5363\times 10^{-4}$	$5.0730\times 10^{-4}$	$4.5381\times 10^{-4}$	$5.0751\times 10^{-4}$
	5	5	$8.3927\times 10^{-5}$	$6.3752\times 10^{-5}$	$8.4069\times 10^{-5}$	$6.4048\times 10^{-5}$	$8.5921\times 10^{-5}$	$6.3155\times 10^{-5}$
	6	6	$5.4065\times 10^{-6}$	$5.7895\times 10^{-6}$	$5.3997\times 10^{-6}$	$5.7975\times 10^{-6}$	$5.4007\times 10^{-6}$	$5.8131\times 10^{-6}$
	7	7	$1.1273\times 10^{-6}$	$8.4051\times 10^{-7}$	$1.1239\times 10^{-6}$	$8.5039\times 10^{-7}$	$1.1503\times 10^{-6}$	$8.4786\times 10^{-7}$

Example 3 Consider the system provided in (2) with $\theta =2,\sigma_{1}=1,\sigma_{2}=2,\eta_{1}(x)=\eta_{2}(x)=x$ and

$$\begin{array}{c} g(x,t)=e^{-\lambda \;t}t^{3}\left(-\frac{6t^{-\alpha }}{\Gamma (4-\alpha )}\cos(x)+e^{-2\;\lambda \;t}t^{6}\sin\left(x\right)\left(2t^{2}+1\right)+\mathrm{xt}\cos\left(x\right)+(x-2)\sin\left(x\right)\right)\\ +i\left(e^{-\lambda \;t}t^{3}\left(\frac{6t^{-\alpha }}{\Gamma (4-\alpha )}\sin(x)+e^{-2\lambda t}t^{6}\cos(x)\left(2t^{2}+1\right)+\mathrm{xt}\sin\left(x\right)+(x-2)\cos\left(x\right)\right)\right),\\ h(x,t)=e^{-\lambda \;t}t^{3}\left(-\frac{24t^{1-\alpha }}{\Gamma (5-\alpha )}\sin(x)+e^{-2\;\lambda \;t}t^{7}\cos\left(x\right)\left(2t^{2}+1\right)+\mathrm{xt}\cos\left(x\right)+x\sin\left(x\right)-2\;t\cos\left(x\right)\right)\\ +i\left(e^{-\lambda \;t}t^{3}\left(\frac{24t^{1-\alpha }}{\Gamma (5-\alpha )}\cos(x)+e^{-2\;\lambda \;t}t^{7}\sin\left(x\right)\left(2t^{2}+1\right)+\mathrm{xt}\sin\left(x\right)-2\;t\sin\left(x\right)+x\cos\left(x\right)\right)\right), \end{array}$$

where

$${\left(\Psi \right|}_{t=0}={\left(\Phi \right|}_{t=0}=0,$$

and

$$\begin{array}{cc} {\left(\Psi \right|}_{x=0}={\mathrm{it}}^{3}e^{-\lambda t}, & {\left(\Psi \right|}_{x=1}=t^{3}e^{-\lambda t}\left(\sin(1)+i\cos(1)\right),\\ {\left(\Phi \right|}_{x=0}=t^{4}e^{-\lambda t}, & {\left(\Phi \right|}_{x=1}=t^{4}e^{-\lambda t}e^{i}\text{.} \end{array}$$

**Fig. 5:**
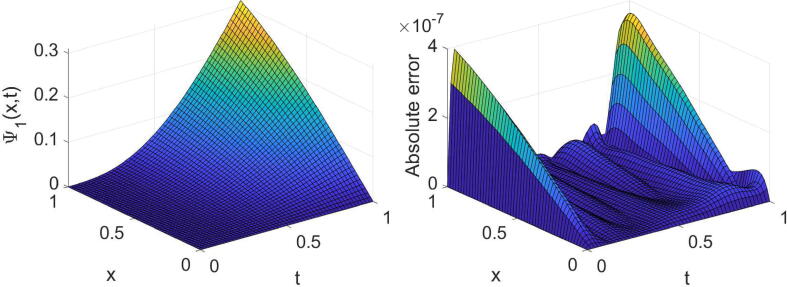
Plots of the obtained solution $\Psi_{1}$ and associated error with α=3/4,λ=1 and (m=6,n=7) in Exa.mple 3.

**Fig. 6:**
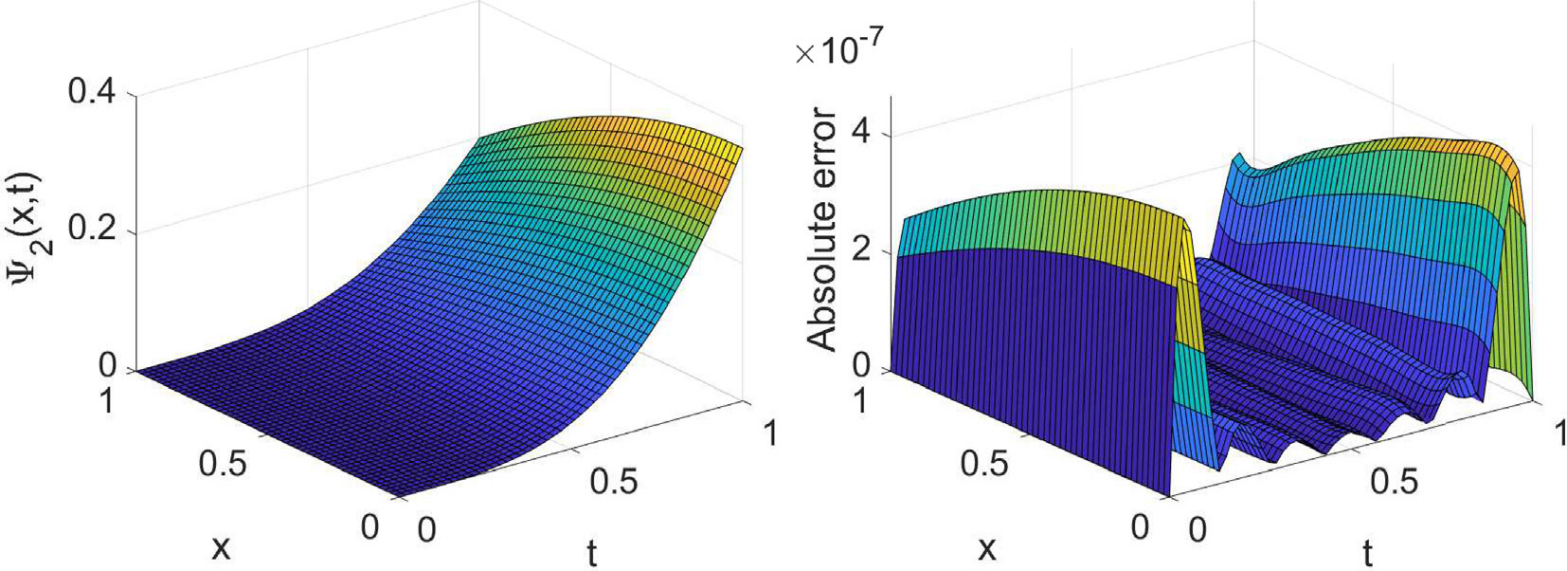
Plots of the obtained solution $\Psi_{2}$ and associated error with α=3/4,λ=1 and (m=6,n=7) in Exa.mple 3.

**Fig. 7:**
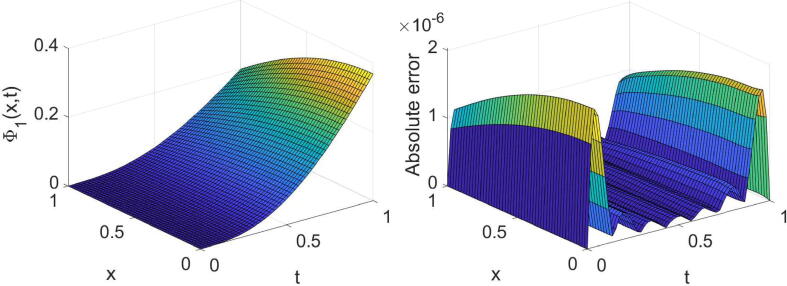
Plots of the obtained solution $\Phi_{1}$ and associated error with α=3/4,λ=1 and (m=6,n=7) in Exa.mple 3.

**Fig. 8:**
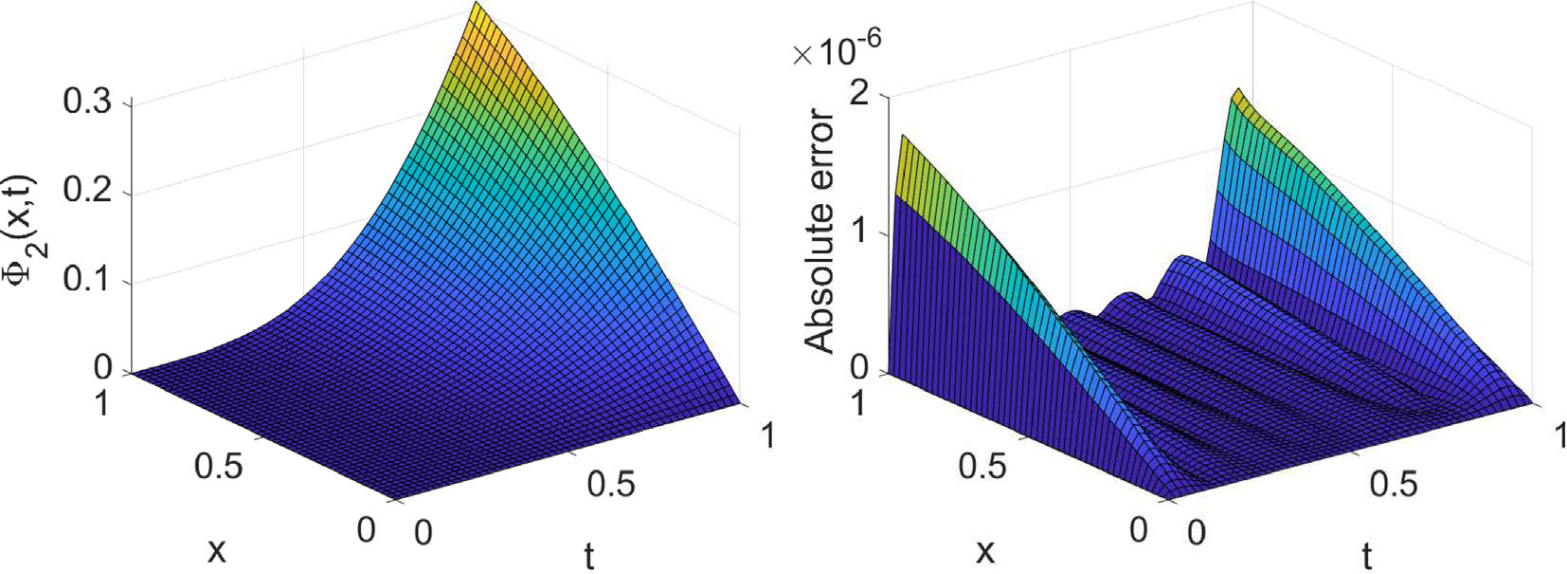
Plots of the obtained solution $\Phi_{2}$ and associated error with α=3/4,λ=1 and (m=6,n=7) in Exa.mple 3.

The system’s exact solution is $\left\{\Psi (x,t)=t^{3}e^{-\lambda t}\left(\sin(x)+i\cos(x)\right),\Phi (x,t)=t^{4}e^{-\lambda t+\mathrm{ix}}\right\}$. The outcomes derived for this system with three values of α, two values of λ and some values of (m,n) are reported in Table 3, Table 4. These tables acknowledge the high accuracy and high convergence of the results. For the specific case, α=3/4,λ=1 and (m=6,n=7), the obtained results are shown graphically in Fig. 5, Fig. 6, Fig. 7, Fig. 8.

**Table 3:** The errors obtained by the established algorithm with some values of (m,n) in Example 3 for Ψ with some choices of α and λ.

			α					
			1/4		1/2		3/4	
λ	*m*	*n*	$e_{\Psi_{1}}$	$e_{\Psi_{2}}$	$e_{\Psi_{1}}$	$e_{\Psi_{2}}$	$e_{\Psi_{1}}$	$e_{\Psi_{2}}$
1	3	4	$2.1155\times 10^{-4}$	$3.6897\times 10^{-4}$	$2.0901\times 10^{-4}$	$3.7004\times 10^{-4}$	$2.0577\times 10^{-4}$	$3.7132\times 10^{-4}$
	4	5	$1.8051\times 10^{-5}$	$2.9759\times 10^{-5}$	$1.8043\times 10^{-5}$	$2.9762\times 10^{-5}$	$1.8033\times 10^{-5}$	$2.9766\times 10^{-5}$
	5	6	$1.2867\times 10^{-6}$	$2.0706\times 10^{-6}$	$1.2884\times 10^{-6}$	$2.0704\times 10^{-6}$	$1.2908\times 10^{-6}$	$2.0701\times 10^{-6}$
	6	7	$8.9157\times 10^{-8}$	$1.3808\times 10^{-7}$	$8.9193\times 10^{-8}$	$1.3805\times 10^{-7}$	$8.9239\times 10^{-8}$	$1.3803\times 10^{-7}$
2	3	4	$2.4778\times 10^{-4}$	$4.1019\times 10^{-4}$	$2.4727\times 10^{-4}$	$4.1044\times 10^{-4}$	$2.4665\times 10^{-4}$	$4.1076\times 10^{-4}$
	4	5	$5.8977\times 10^{-5}$	$9.6717\times 10^{-5}$	$5.8974\times 10^{-5}$	$9.6718\times 10^{-5}$	$5.8969\times 10^{-5}$	$9.6720\times 10^{-5}$
	5	6	$9.8481\times 10^{-6}$	$1.6062\times 10^{-5}$	$9.8498\times 10^{-6}$	$1.6061\times 10^{-5}$	$9.8519\times 10^{-6}$	$1.6060\times 10^{-5}$
	6	7	$1.2949\times 10^{-6}$	$2.1132\times 10^{-6}$	$1.2949\times 10^{-6}$	$2.1132\times 10^{-6}$	$1.2949\times 10^{-6}$	$2.1132\times 10^{-6}$

**Table 4:** The errors obtained by the established algorithm with some values of (m,n) in Example 3 for Φ with some choices of α and λ.

			α					
			1/4		1/2		3/4	
λ	*m*	*n*	$e_{\Phi_{1}}$	$e_{\Phi_{2}}$	$e_{\Phi_{1}}$	$e_{\Phi_{2}}$	$e_{\Phi_{1}}$	$e_{\Phi_{2}}$
1	3	4	$2.6945\times 10^{-4}$	$1.6772\times 10^{-4}$	$2.6896\times 10^{-4}$	$1.6862\times 10^{-4}$	$2.6827\times 10^{-4}$	$1.6998\times 10^{-4}$
	4	5	$6.2078\times 10^{-5}$	$3.8950\times 10^{-5}$	$6.2090\times 10^{-5}$	$3.8935\times 10^{-5}$	$6.2107\times 10^{-5}$	$3.8914\times 10^{-5}$
	5	6	$7.3889\times 10^{-6}$	$4.5267\times 10^{-6}$	$7.3824\times 10^{-6}$	$4.5337\times 10^{-6}$	$7.3728\times 10^{-6}$	$4.5431\times 10^{-6}$
	6	7	$5.8870\times 10^{-7}$	$3.5817\times 10^{-7}$	$5.8868\times 10^{-7}$	$3.5820\times 10^{-7}$	$5.8864\times 10^{-7}$	$3.5824\times 10^{-7}$
2	3	4	$1.4466\times 10^{-4}$	$8.9891\times 10^{-5}$	$1.4363\times 10^{-4}$	$9.1049\times 10^{-5}$	$1.4213\times 10^{-4}$	$9.2613\times 10^{-5}$
	4	5	$6.0039\times 10^{-5}$	$3.6949\times 10^{-5}$	$6.0041\times 10^{-5}$	$3.6946\times 10^{-5}$	$6.0044\times 10^{-5}$	$3.6942\times 10^{-5}$
	5	6	$1.9633\times 10^{-5}$	$1.2021\times 10^{-5}$	$1.9632\times 10^{-5}$	$1.2023\times 10^{-5}$	$1.9629\times 10^{-5}$	$1.2025\times 10^{-5}$
	6	7	$3.7359\times 10^{-6}$	$2.2866\times 10^{-6}$	$3.7359\times 10^{-6}$	$2.2866\times 10^{-6}$	$3.7359\times 10^{-6}$	$2.2867\times 10^{-6}$

Example 4 Consider the system introduced in (2) with $\theta =1,\sigma_{1}=\sigma_{2}=1/2,\eta_{1}(x)=\cos(x),\eta_{2}(x)=\sin(x)$ and

$$\begin{array}{c} g(x,t)=-t^{1-\alpha }e^{-(\lambda t+x)}E_{2,2-\alpha }\left(-t^{2}\right)+e^{-(\lambda t+x)}\cos\left(t\right)\left(e^{-2(\lambda t+x)}+\cos\left(x\right)+\sin\left(x\right)+1\right)\\ +i\left(-t^{2-\alpha }e^{-(\lambda t+x)}E_{2,3-\alpha }\left(-t^{2}\right)+e^{-(\lambda t+x)}\sin\left(t\right)\left(e^{-2(\lambda t+x)}+\cos\left(x\right)-\sin\left(x\right)+1\right)\right),\\ h(x,t)=t^{1-\alpha }e^{-(\lambda t+x)}E_{2,2-\alpha }\left(-t^{2}\right)+e^{-(\lambda t+x)}\cos\left(t\right)\left(e^{-2(\lambda t+x)}+\cos\left(x\right)+\sin\left(x\right)+1\right)\\ +i\left(-t^{2-\alpha }e^{-(\lambda t+x)}E_{2,3-\alpha }\left(-t^{2}\right)-e^{-(\lambda t+x)}\sin\left(t\right)\left(e^{-2(\lambda t+x)}+\cos\left(x\right)-\sin\left(x\right)+1\right)\right), \end{array}$$

where

$${\left(\Psi \right|}_{t=0}={\left(\Phi \right|}_{t=0}=e^{-x},$$

and

$$\begin{array}{cc} {\left(\Psi \right|}_{x=0}=e^{(i-\lambda )t}, & {\left(\Psi \right|}_{x=1}=e^{(i-\lambda )t-1},\\ {\left(\Phi \right|}_{x=0}=e^{-(i+\lambda )t}, & {\left(\Phi \right|}_{x=1}=e^{-(i+\lambda )t-1}\text{.} \end{array}$$

**Fig. 9:**
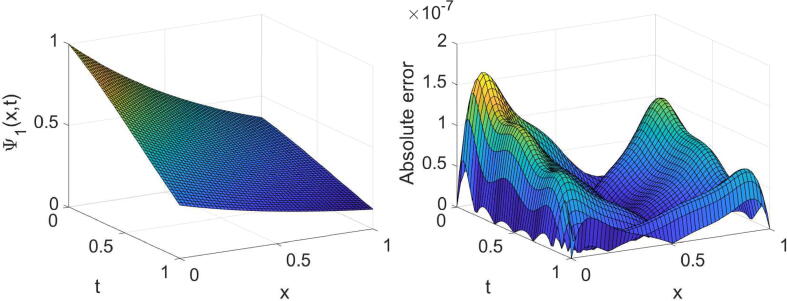
Plots of the obtained solution $\Psi_{1}$ and associated error with α=1/4,λ=1/2 and (m=n=6) in Exa.mple 4.

**Fig. 10:**
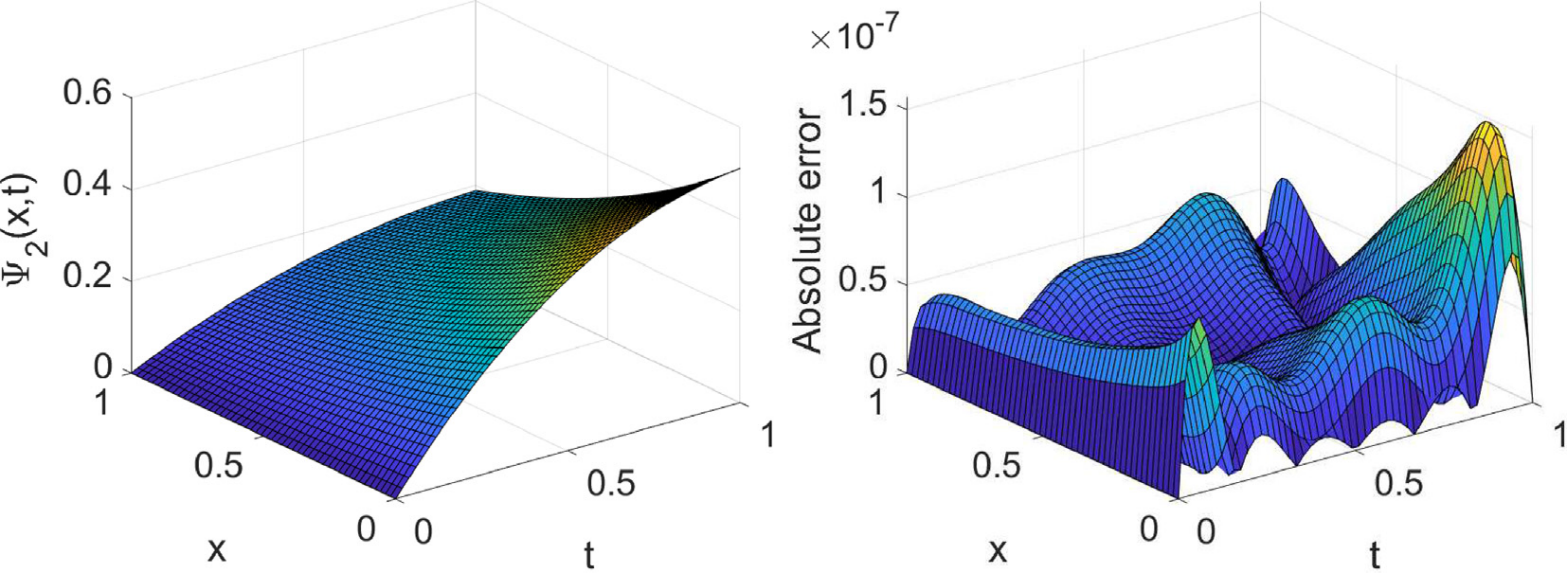
Plots of the obtained solution $\Psi_{2}$ and associated error with α=1/4,λ=1/2 and (m=n=6) in Exa.mple 4.

**Fig. 11:**
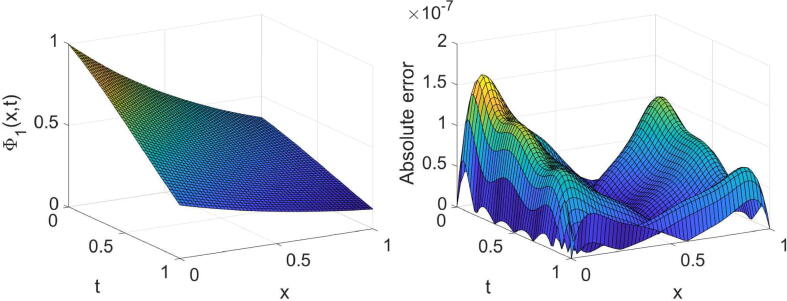
Plots of the obtained solution $\Phi_{1}$ and associated error with α=1/4,λ=1/2 and (m=n=6) in Exa.mple 4.

**Fig. 12:**
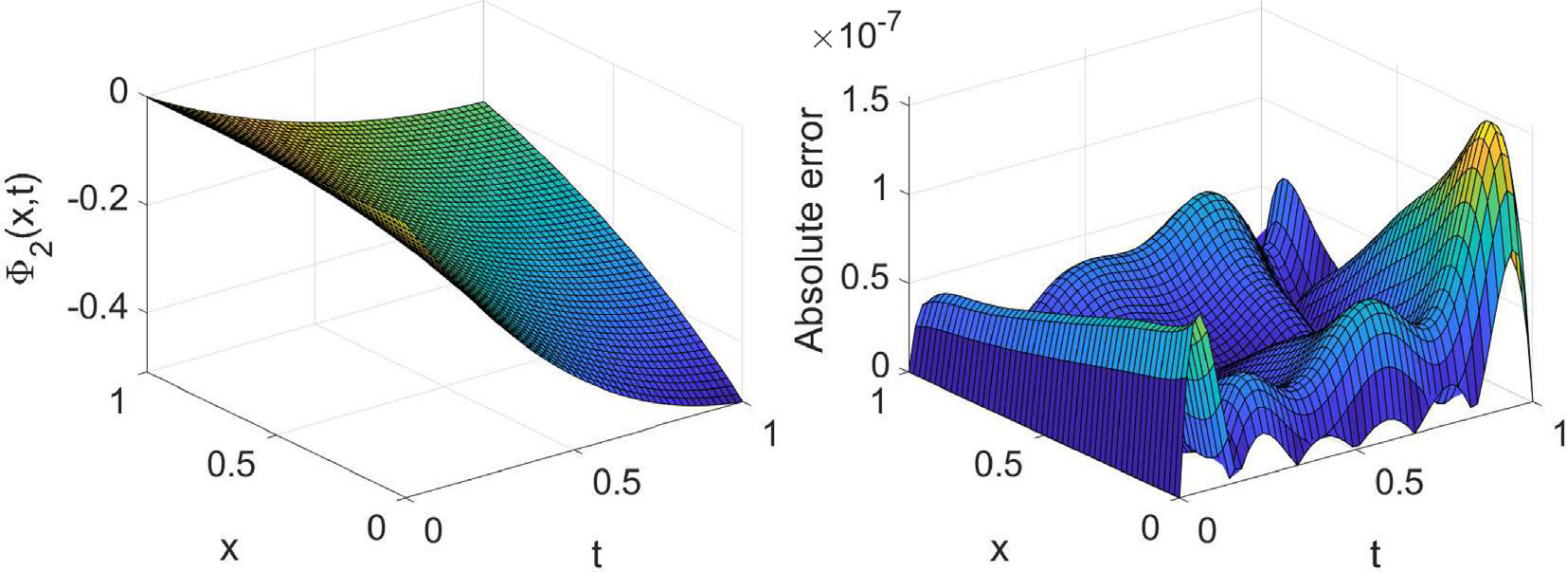
Plots of the obtained solution $\Phi_{2}$ and associated error with α=1/4,λ=1/2 and (m=n=6) in Exa.mple 4.

The system’s exact solution is $\left\{\Psi (x,t)=e^{(i-\lambda )t-x},\Phi (x,t)=e^{-(i+\lambda )t-x}\right\}$. The desired method is applied with different values of (m,n) for this system with three values of α and two values of λ. The extracted results are listed in Table 5, Table 6. These tables acknowledge the high accuracy and high convergence of the results. For the specific case, α=1/4,λ=1/2 and (m=n=6), the numerical results are illustrated graphically in Fig. 9, Fig. 10, Fig. 11, Fig. 12.

**Table 5:** The errors obtained by the established algorithm with some values of (m,n) in Example 4 for Ψ with some choices of α and λ.

			α					
			1/4		1/2		3/4	
λ	*m*	*n*	$e_{\Psi_{1}}$	$e_{\Psi_{2}}$	$e_{\Psi_{1}}$	$e_{\Psi_{2}}$	$e_{\Psi_{1}}$	$e_{\Psi_{2}}$
1/2	3	3	$4.0345\times 10^{-4}$	$3.7615\times 10^{-4}$	$4.0290\times 10^{-4}$	$3.6293\times 10^{-4}$	$4.0119\times 10^{-4}$	$3.4473\times 10^{-4}$
	4	4	$1.7186\times 10^{-5}$	$1.5267\times 10^{-5}$	$1.6874\times 10^{-5}$	$1.5014\times 10^{-5}$	$1.6806\times 10^{-5}$	$1.4882\times 10^{-5}$
	5	5	$2.3134\times 10^{-6}$	$1.3353\times 10^{-6}$	$2.2944\times 10^{-6}$	$1.3283\times 10^{-6}$	$2.4188\times 10^{-6}$	$1.3591\times 10^{-6}$
	6	6	$5.8657\times 10^{-8}$	$4.1996\times 10^{-8}$	$5.9339\times 10^{-8}$	$4.1324\times 10^{-8}$	$6.3781\times 10^{-8}$	$4.1615\times 10^{-8}$
1	3	3	$4.7877\times 10^{-4}$	$2.5291\times 10^{-4}$	$4.7666\times 10^{-4}$	$2.4895\times 10^{-4}$	$4.7165\times 10^{-4}$	$2.4040\times 10^{-4}$
	4	4	$3.5514\times 10^{-5}$	$1.2699\times 10^{-5}$	$3.5715\times 10^{-5}$	$1.2466\times 10^{-5}$	$3.6209\times 10^{-5}$	$1.2276\times 10^{-5}$
	5	5	$1.7140\times 10^{-6}$	$2.4001\times 10^{-6}$	$1.7053\times 10^{-6}$	$2.4370\times 10^{-6}$	$1.7918\times 10^{-6}$	$2.5271\times 10^{-6}$
	6	6	$6.2314\times 10^{-8}$	$1.2027\times 10^{-7}$	$6.3413\times 10^{-8}$	$1.2113\times 10^{-7}$	$6.9010\times 10^{-8}$	$1.2095\times 10^{-8}$

**Table 6:** The errors obtained by the established algorithm with some values of (m,n) in Example 4 for Φ with some choices of α and λ.

			α					
			1/4		1/2		3/4	
λ	*m*	*n*	$e_{\Phi_{1}}$	$e_{\Phi_{2}}$	$e_{\Phi_{1}}$	$e_{\Phi_{2}}$	$e_{\Phi_{1}}$	$e_{\Phi_{2}}$
1/2	3	3	$4.4547\times 10^{-4}$	$3.1492\times 10^{-4}$	$4.4899\times 10^{-4}$	$3.2801\times 10^{-4}$	$4.4748\times 10^{-4}$	$3.5140\times 10^{-4}$
	4	4	$1.8037\times 10^{-5}$	$1.4830\times 10^{-5}$	$1.8554\times 10^{-5}$	$1.4968\times 10^{-5}$	$3.5205\times 10^{-5}$	$1.3312\times 10^{-5}$
	5	5	$2.5500\times 10^{-6}$	$9.8965\times 10^{-7}$	$2.6158\times 10^{-6}$	$1.0818\times 10^{-6}$	$2.8466\times 10^{-6}$	$1.3206\times 10^{-6}$
	6	6	$5.9470\times 10^{-8}$	$4.1178\times 10^{-8}$	$5.9209\times 10^{-8}$	$4.2056\times 10^{-8}$	$6.0356\times 10^{-8}$	$4.7575\times 10^{-8}$
1	3	3	$4.9271\times 10^{-4}$	$2.2185\times 10^{-4}$	$4.9384\times 10^{-4}$	$2.2069\times 10^{-4}$	$4.9101\times 10^{-4}$	$2.2037\times 10^{-4}$
	4	4	$3.5549\times 10^{-5}$	$1.2335\times 10^{-5}$	$3.5366\times 10^{-5}$	$1.2505\times 10^{-5}$	$1.9596\times 10^{-5}$	$1.5556\times 10^{-5}$
	5	5	$1.8534\times 10^{-6}$	$2.1724\times 10^{-7}$	$1.9051\times 10^{-6}$	$2.1581\times 10^{-6}$	$2.0848\times 10^{-6}$	$2.1801\times 10^{-6}$
	6	6	$6.2219\times 10^{-8}$	$1.1880\times 10^{-7}$	$6.1362\times 10^{-8}$	$1.1800\times 10^{-8}$	$6.1219\times 10^{-8}$	$1.1441\times 10^{-7}$

## Conclusion

In this paper, the tempered fractional derivative in the Caputo form was utilized to define the time fractional nonlinear Schrödinger equation and a coupled system of nonlinear Schrödinger equations. The ODCPs were used as basis functions to design the collocation strategy for these problems. To this aim, some matrix relationships for these polynomials were obtained. In the designed procedures, the problem’s solution were obtained by solving an algebraic system of equations. These systems were obtained by approximating the solution with the ODCPs and employing the expressed matrix relationships, along with the collocation technique. Some examples were presented to check the validity of the developed algorithms. The reported results acknowledged the high accuracy of the designed schemes. As a future research direction, the strategy presented in this study can be developed for the similar problems in higher dimensional. In addition, the established method can be used for the tempered fractional version of different problems, such as the ones investigated in [55], [56], [57], [58], [59].

## Compliance with Ethics Requirements

This paper does not contain any studies with human or animal subjects.

## CREDIT Author Statement

**Mohammad Hossein Heydari** Conceptualization, Methodology, Formal analysis, Investigation, Validation, Writing-original draft, Writing-review & editing. **Dumitru Baleanu** Investigation, Validation, Writing-review & editing.

## Declaration of Competing Interest

The authors declare that they have no known competing financial interests or personal relationships that could have appeared to influence the work reported in this paper.
